# A spectrum of altered non-rapid eye movement sleep in schizophrenia

**DOI:** 10.1093/sleep/zsae218

**Published:** 2024-09-19

**Authors:** Nataliia Kozhemiako, Chenguang Jiang, Yifan Sun, Zhenglin Guo, Sinéad Chapman, Guanchen Gai, Zhe Wang, Lin Zhou, Shen Li, Robert G Law, Lei A Wang, Dimitrios Mylonas, Lu Shen, Michael Murphy, Shengying Qin, Wei Zhu, Zhenhe Zhou, Robert Stickgold, Hailiang Huang, Shuping Tan, Dara S Manoach, Jun Wang, Mei-Hua Hall, Jen Q Pan, Shaun M Purcell

**Affiliations:** Department of Psychiatry, Brigham and Women’s Hospital, Harvard Medical School, Boston, MA, USA; Department of Psychiatry, The Affiliated Mental Health Center of Jiangnan University, Wuxi Central Rehabilitation Hospital, Wuxi, China; Department of Psychiatry, The Affiliated Mental Health Center of Jiangnan University, Wuxi Central Rehabilitation Hospital, Wuxi, China; Stanley Center for Psychiatric Research, Broad Institute of MIT and Harvard, Boston, MA, USA; Stanley Center for Psychiatric Research, Broad Institute of MIT and Harvard, Boston, MA, USA; Department of Psychiatry, The Affiliated Mental Health Center of Jiangnan University, Wuxi Central Rehabilitation Hospital, Wuxi, China; Department of Psychiatry, The Affiliated Mental Health Center of Jiangnan University, Wuxi Central Rehabilitation Hospital, Wuxi, China; Stanley Center for Psychiatric Research, Broad Institute of MIT and Harvard, Boston, MA, USA; Department of Psychiatry, McLean Hospital, Harvard Medical School, Boston, MA, USA; Department of Psychiatry, Brigham and Women’s Hospital, Harvard Medical School, Boston, MA, USA; Stanley Center for Psychiatric Research, Broad Institute of MIT and Harvard, Boston, MA, USA; Department of Psychiatry, Massachusetts General Hospital, Harvard Medical School, Boston, MA, USA; Key Laboratory for the Genetics of Developmental and Neuropsychiatric Disorders (Ministry of Education), Bio-X Institutes, Shanghai Jiao Tong University, Shanghai, China; Department of Psychiatry, McLean Hospital, Harvard Medical School, Boston, MA, USA; Key Laboratory for the Genetics of Developmental and Neuropsychiatric Disorders (Ministry of Education), Bio-X Institutes, Shanghai Jiao Tong University, Shanghai, China; Department of Psychiatry, The Affiliated Mental Health Center of Jiangnan University, Wuxi Central Rehabilitation Hospital, Wuxi, China; Department of Psychiatry, The Affiliated Mental Health Center of Jiangnan University, Wuxi Central Rehabilitation Hospital, Wuxi, China; Department of Psychiatry, Beth Israel Deaconess Medical Center, Boston, MA, USA; Department of Psychiatry, Harvard Medical School, Boston, MA, USA; Stanley Center for Psychiatric Research, Broad Institute of MIT and Harvard, Boston, MA, USA; ATGU, MGH, Harvard Medical School, Boston, MA, USA; Psychiatry Research Center, Peking University Huilongguan Clinical Medical School, Beijing Huilongguan Hospital, Beijing University, Beijing, China; Department of Psychiatry, Massachusetts General Hospital, Harvard Medical School, Boston, MA, USA; Department of Psychiatry, The Affiliated Mental Health Center of Jiangnan University, Wuxi Central Rehabilitation Hospital, Wuxi, China; Department of Psychiatry, McLean Hospital, Harvard Medical School, Boston, MA, USA; Stanley Center for Psychiatric Research, Broad Institute of MIT and Harvard, Boston, MA, USA; Department of Psychiatry, Brigham and Women’s Hospital, Harvard Medical School, Boston, MA, USA; Department of Psychiatry, Harvard Medical School, Boston, MA, USA

**Keywords:** *sleep spindles*, *biomarkers*, *EEG analysis*, *psychiatric disorders*

## Abstract

Multiple facets of sleep neurophysiology, including electroencephalography (EEG) metrics such as non-rapid eye movement (NREM) spindles and slow oscillations, are altered in individuals with schizophrenia (SCZ). However, beyond group-level analyses, the extent to which NREM deficits vary among patients is unclear, as are their relationships to other sources of heterogeneity including clinical factors, aging, cognitive profiles, and medication regimens. Using newly collected high-density sleep EEG data on 103 individuals with SCZ and 68 controls, we first sought to replicate our previously reported group-level differences between patients and controls (original *N* = 130) during the N2 stage. Then in the combined sample (*N* = 301 including 175 patients), we characterized patient-to-patient variability. We replicated all group-level mean differences and confirmed the high accuracy of our predictive model (area under the receiver operating characteristic curve [AUC] = 0.93 for diagnosis). Compared to controls, patients showed significantly increased between-individual variability across many (26%) sleep metrics. Although multiple clinical and cognitive factors were associated with NREM metrics, collectively they did not account for much of the general increase in patient-to-patient variability. The medication regimen was a greater contributor to variability. Some sleep metrics including fast spindle density showed exaggerated age-related effects in SCZ, and patients exhibited older predicted biological ages based on the sleep EEG; further, among patients, certain medications exacerbated these effects, in particular olanzapine. Collectively, our results point to a spectrum of N2 sleep deficits among SCZ patients that can be measured objectively and at scale, with relevance to both the etiological heterogeneity of SCZ as well as potential iatrogenic effects of antipsychotic medication.

## Graphical Abstract

**Graphical Abstract:**
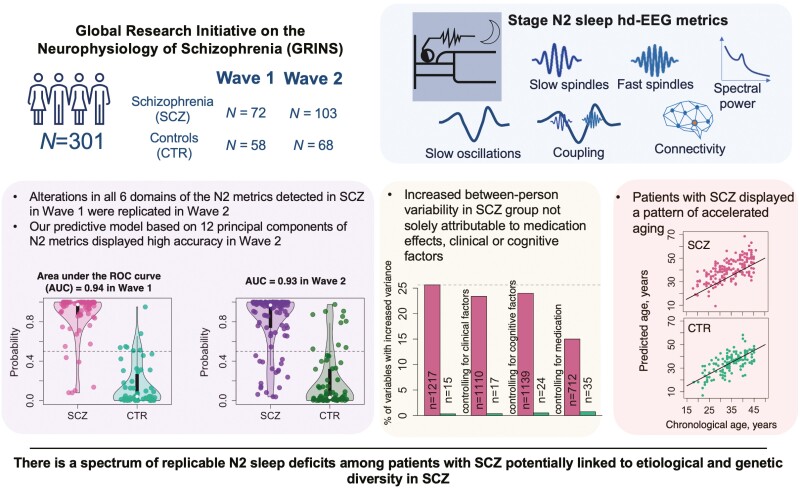

**Significance of Statement**

Sleep neurophysiology, particularly non-rapid eye movement (NREM) fast spindles and slow oscillations, is altered in individuals with schizophrenia (SCZ). Here, we confirmed group-level differences and additionally identified increased patient-to-patient variability in many NREM metrics, which was largely independent of clinical and cognitive differences. In contrast, medication regimens significantly contributed to this variability. SCZ patients showed exacerbated age-related effects in certain sleep metrics, suggesting an accelerated biological aging process, albeit one that may in part reflect the adverse effects of antipsychotics. These findings underscore the diversity of NREM deficits in SCZ, providing insights into its etiological diversity, treatment response, and prognosis.

Schizophrenia (SCZ) is a heterogeneous neuropsychiatric disorder characterized by variable combinations of positive and negative symptoms and cognitive deficits. It is highly heritable and polygenic [1, 2] and has a substantial impact on affected individuals and their caregivers [3]. One of the most urgent tasks in SCZ research is the identification of objective biomarkers for neurobiological deficits to aid in diagnostics, prognostics, patient stratifications, and to guide novel therapeutic approaches. A growing body of literature [4–6], including our own findings [7], points to sleep neurophysiology as providing a rich array of putative electroencephalography (EEG) biomarkers with robust and replicable group differences between patients with SCZ and healthy controls. Notably, we previously showed that sleep-based biomarkers were largely independent of wake EEG metrics from event-related potential (ERP) paradigms [8–10] measured in the same individuals, suggesting the sleep EEG offers unique information about the neural underpinnings of SCZ.

However, a biomarker that only tracks with current diagnostic status is arguably of limited value. For a heterogeneous disease such as SCZ, biomarkers that support clinically meaningful stratification of patients, for example, by etiology (neurobiological deficits) or likelihood of treatment response, are much needed. We posited that person-to-person variability in sleep micro-architecture may index etiologically relevant heterogeneity among patients. As group-level mean differences tend to reflect only commonalities among patients, biomarkers exhibiting increased patient-specific variability may be more likely to reflect distinct continua of risk, heterogeneous subtypes of disease pathophysiology, or differential course and response to treatment.

In this study, we therefore searched for sleep-architecture metrics showing increased between-person variability in patients, alongside the standard assessment of differences in group means. We next related the sleep metrics to other measures known to vary among patients, namely clinical symptoms, cognitive deficits, and illness duration. Whereas an emerging literature points to replicable group-level alterations in sleep physiology in SCZ [6], the broader landscape—of heterogeneity among SCZ patients as well as specificity across other neurological and psychiatric diseases—is less well charted. A recent review focused on sleep spindles, symptomatology, and cognitive deficits in SCZ concluded that small sample sizes and inconsistent methodologies led to a high risk of bias and deterred strong conclusions [11]. Despite some support for associations between spindles and attention/cognitive processing speed in patients and the role of spindles in memory consolidation [12], robust connections between non-rapid eye movement (NREM) sleep and SCZ symptomatology have not been well established [13].

Patient-to-patient variation in sleep architecture may also be attributable to different medication regimens. Psychoactive drugs significantly impact sleep patterns as well as sleep EEG [14], although associations can be complex: they can normalize some aspects of sleep in SCZ but disrupt others [15]. Furthermore, a growing literature points to adverse cognitive side effects of antipsychotic medication, likely reflecting anticholinergic burden [16–18], which is particularly relevant given reported links between NREM sleep and cognitive performance [11, 12]. While studies of unmedicated patients with SCZ have established that sleep abnormalities (notably, spindle deficits) persist independent of medication [19], recent reviews have pointed to the need for research to better characterize the roles of medications in generating sleep alterations [20, 21]. In particular, larger sample sizes—such as those offered by the current study—will be necessary to resolve the impact of different medications on sleep architecture in patients and ultimately to disentangle it from underlying disease-associated signatures.

Finally, beyond illness duration per se, variation among patients may reflect the differential effects of aging. Substantial imaging literature has pointed to accelerated brain aging in SCZ [22, 23], which may in part account for its increased burden of age-related morbidity and premature mortality [24]. The NREM EEG changes profoundly across typical development [25] and delayed or accelerated patterns have also been shown to predict diverse pathologies [26, 27]. We therefore also analyzed the sleep EEG data through the lens of biological/brain age prediction to address the hypothesis of accelerated brain aging in SCZ.

Here we report on our ongoing Global Research Initiative of Neurophysiology on Schizophrenia (GRINS) study, in particular the independent second wave of *N* = 103 SCZ patients and *N* = 68 controls with overnight high-density EEG and extensive clinical, cognitive, and demographic data. To establish whether sleep macro- and micro-architecture provide a robust and novel window on SCZ heterogeneity, we first sought to replicate our previously reported group-level mean differences in sleep physiology [7]. Subsequently, in the combined sample (*N* = 301) we determined whether between-individual variability in the same set of metrics was altered in SCZ. Finally, we investigated whether differences could be explained by measured clinical and cognitive factors, illness duration and aging, or medication use (see Supplementary Figure S1 for a schematic illustration of the study design).

## Methods

### Participants

Data on 301 individuals (175 SCZ and 126 healthy controls [CTR, see the full list of abbreviations in Supplementary Table S1]) were collected as part of the GRINS study (Table 1). A portion of these data (*N* = 130, wave 1) was collected before October 12, 2020, and was used in our prior report [7]. Since then, the second wave of data (171 additional individuals, wave 2) was collected following the same protocol and inclusion–exclusion criteria as described in [7]. In brief, all participants were aged 18–45 with normal IQ (>70). Patients with schizophrenia or schizoaffective disorder were recruited from Wuxi Mental Health Center and diagnosed according to DSM-5. Control participants, without any mental disorders or a family history thereof, were recruited from the local community through advertisements. Additionally, the following exclusion criteria applied to all participants: (1) less than 6 months since electroconvulsive treatment, (2) self-reported sleep disorders or barbiturate use, (3) severe medical conditions like epilepsy or head injury, (4) hearing impairment (above 45 dB at 1000 Hz), and (5) pregnancy or lactation. With regard to sleep disorders, in addition to self-reported frequent difficulty in falling asleep and waking up easily during the night, the exclusion criteria also included a diagnosed sleep disorder (e.g. restless leg syndrome) based on chart review, and a STOP-BANG score of 4 or above, indicating a high risk of obstructive sleep apnea–hypopnea syndrome. Only one patient with SCZ was excluded due to present sleep disorders during the recruitment process. Informed written consent was given by all participants. The study conformed to the Declaration of Helsinki and was approved by the Harvard TH Chan School of Public Health Office of Human Research Administration (IRB18-0058) as well as the Institutional Review Board of WMHC (WXMHCIRB2018LLKY003).

**Table 1.** Key Demographic and Clinical Variables Stratified by Wave

*Characteristics*	*Wave 1*		Wave 2		Combined	
	*SCZ*	*CTR*	*SCZ*	*CTR*	*SCZ*	*CTR*
*N*	72	58	103	68	175	126
*Sex, females (%)*	25 (35%)	22 (31%)	49 (48%)	31 (30%)	74 (42%)	53 (30%)
*Age, years*	35 ± 7	**32 ± 6.3**	34 ± 7.5	**36 ± 6** ^†^	35 ± 7.3	34 ± 6.5
*Maternal education, higher than middle school (%)*	30 (42%)	14 (24%)	29 (28%)	21 (31%)	59 (34%)	35 (28%)
*Paternal education, higher than middle school (%)*	31 (43%)	22 (38%)	37 (36%)	21 (31%)	68 (39%)	43 (34%)
*Illness duration, years*	12 ± 7.0		10 ± 6.9		11 ± 7.0	
*PANSS positive*	15 ± 5.8^‡^		17 ± 6.3^‡^		16 ± 6.1^‡^	
*PANSS negative*	16 ± 5.7^‡^		18 ± 6.7^‡^		17 ± 6.4^‡^	
*PANSS general*	33 ± 9^‡^		35 ± 10.9^‡^		34 ± 10.1^‡^	
*SCZ duration, years*	12 ± 7		10 ± 6.6		11 ± 7	
*MCCB total*	43 ± 9.9		44 ± 8.7		44 ± 9.2	
*Equivalent antipsychotic dose, mg*	612 ± 262.8		676 ± 244.6		649 ± 253.6	
*Time in bed, mins*	**532 ± 55.7** ^*^	**467 ± 44.5**	**540 ± 43.9** ^*^	**456 ± 27.7**	**537 ± 47.8** ^*^	**461 ± 36.8**
*Total sleep time, mins*	378 ± 95.1	382 ± 63.1	398 ± 84.2	377 ± 48.1	390 ± 88.9	376 ± 59.4
*Wake after sleep onset, mins*	65 ± 44.1	49 ± 34.4	70 ± 46.8	55 ± 39.7	**67 ± 43.9** ^*^	**52 ± 37.4**
*Sleep efficiency (TST/TIB)*	**73 ± 14.1** ^*^	**82 ± 10.9**	**74 ± 13.8** ^*^	**83 ± 9.5**	**74 ± 13.8** ^*^	**82 ± 10.1**
*Sleep maintenance efficiency (TST/start-end of sleep)*	81 ± 13.8	86 ± 10.2	85 ± 9.8	87 ± 8.7	**83 ± 11.9** ^*^	**87 ± 9.4**
*N1 duration, mins (%)*	42 ± 26.2 (11%)	32 ± 17.3 (9%)	33 ± 16.8 (9%)	33 ± 16.5 (9%)	37 ± 20.4 (10%)	32 ± 16.8 (9%)
*N2 duration, mins (%)*	186 ± 69.8 (50%)	204 ± 48.2 (53%)	209 ± 72.2 (53%)	217 ± 37.7 (57%)	201 ± 73.5 (52%)	211 ± 43.2 (56%)
*N3 duration, mins (%)*	76 ± 49.8 (21%)	**80 ± 31** (21%)	72 ± 48.4 (18%)	**60 ± 26.5** ^†^ (16%)	74 ± 48.9 (19%)	69 ± 30.3 (18%)
*REM duration, mins (%)*	66 ± 37.6 (17%)	65 ± 25.1 (17%)	76 ± 34.4 (19%)	66 ± 22.9 (18%)	72 ± 36.1 (18%)	65 ± 23.8 (17%)
*REM latency, mins*	121 ± 55.2	115 ± 59	112 ± 57.4	97 ± 38.6	116 ± 56.5	105 ± 49.8
*Number of cycles*	4 ± 1.4	4 ± 0.7	4 ± 1.2	4 ± 0.9	4 ± 1.3	4 ± 0.8
*Cycle duration, mins*	99 ± 28.3	104 ± 21.9	97 ± 25.2	93 ± 18.5	98 ± 26.4	98 ± 20.8

^*^differences between SCZ and CTR groups (*p*-value < .01), also highlighted in bold.

^†^differences between waves 1 and 2 within the diagnostic group (*p*-value < .01), also highlighted in bold.

^‡^such scores are considered to represent mild symptoms.

### Data acquisition

All participants underwent three separate visits: (1) to determine eligibility, (2) clinical assessments and the collection of demographic and other medical information, and (3) an overnight EEG, including an ERP session in the evening. The ERP paradigms included sensory gating, auditory 40 Hz steady-state response, and mismatch negativity. EEG recordings used a customized 64-channel EasyCap and the BrainAmp Standard recorder (manufactured by Brain Products GmbH, Germany) at a sampling rate of 500 Hz.

Diagnoses of schizophrenia or schizoaffective disorder were validated using the Structured Clinical Interview for DSM Disorders (SCID) [28]. Control participants were screened by a psychiatrist to confirm the absence of major mental disorders. The collection of clinical information (the Positive and Negative Syndrome Scale [PANSS] [29]) and cognitive assessments (the MATRICS Consensus Cognitive Battery [MCCB]) [30] was conducted by a full-time researcher. PANSS and SCID assessments were conducted by trained psychiatrists, within a week of the sleep EEG. Medication information was collected during the same visit and total antipsychotic dosage was subsequently computed as equivalents of 100 mg of chlorpromazine (CPZ) [31].

### MST task description

The Finger Tapping Motor Sequence Task (MST) involved quickly and accurately pressing four labeled keys on a computer keyboard with the left hand in a 5-element sequence for 30 seconds (a trial). The MST included training and testing runs and was run twice: an overnight session with a training run in the evening (12 trials separated by 30 seconds rest breaks) and an identical testing run in the morning after sleep and a morning control session with training on a new sequence (12 trials) and testing (6 trials) runs in the morning with only 10 minutes in between. A monetary reward was given for total correct sequences to motivate performance similar to previous studies. The primary MST measure was the number of correct sequences per trial, reflecting both speed and accuracy. Overnight improvement was calculated as the percentage increase in correct sequences from the best three training trials to the first three test trials the next morning. For consistency with prior literature, the last three training trials were also used to compute percentage improvement but it did not alter the results. The learning rate during training was computed as an average of the best three training trials divided by the first trial performance.

### Sleep EEG analysis

Sleep staging was performed manually for 30-second epochs by a certified polysomnographic technician using standard AASM criteria, based on C4-M1, F4-M1, O2-M1, all EOG, and EMG channels [32]. An open-source package Luna (http://zzz.bwh.harvard.edu/luna/) developed by us (SMP) was used to process the sleep EEG data. EEG channels were re-referenced to linked mastoids, down-sampled to 200 Hz, and band-pass filtered (0.3–35 Hz). Subsequently, all epochs of a specific stage (here all analyses focused primarily on the N2 sleep stage, as in [7]) had outliers removed or interpolated based on the next steps. Firstly, we detected channels with significant and persistent artifacts. Problematic channels were interpolated using spherical spline interpolation [33]. A channel was designated as bad if over 30% of its data epochs deviated by more than 2 standard deviations from the mean of all channels, in relation to any of the three Hjorth parameters: activity, mobility, and complexity, as originally proposed by [34]. This comparison was made within each epoch across all channels. Secondly, we identified outlier epochs by comparing them to all other epochs recorded from all EEG channels. This comparison was done using the same Hjorth criteria but with a threshold of four standard deviations. Additionally, any epochs with maximum amplitudes exceeding 500 μV, or those exhibiting flat or clipped signals for more than 10% of the epoch’s duration, were also marked as outliers and subsequently interpolated. On average, 7.6% and 7.8% epochs were removed in SCZ and CTR groups, respectively, in wave 2, leaving 386 (43–751) and 400 (92–562) epochs in each group. There were no significant group differences (*p-value *> 0.05) in the proportion of epochs removed or the number of remaining epochs.

### Spindle, SOs detection, and coupling estimation

Spindles were detected using a wavelet method with specific center frequencies of 11 Hz for slow spindles (SS) and 15 Hz for FS targeting approximately +/− 2 Hz. Putative spindles were identified based on exceeding certain thresholds and temporal criteria described in [7]. As an additional quality control procedure, the relative increase in activity in non-spindle bands (delta, theta, and 20–30 Hz beta) was compared to the increase in spindle frequency activity, to exclude non-spindle transients or artifacts that did not primarily reflect sigma-band activity. For passing spindles, we estimated their density, amplitude, integrated spindle activity normalized by spindle count (ISA), duration, observed frequency, and chirp.

SOs were identified by detecting zero-crossings in the 0.3–4 Hz bandpass-filtered EEG signals based on specific temporal criteria (zero-crossing leading to a negative peak was between 0.3 and 1.5 seconds long; a zero-crossing leading to a positive peak was not longer than 1 second) and adaptive/relative amplitude thresholds (twice the size of the signal mean). For each channel, SO density, negative-peak and peak-to-peak amplitude, duration, and upward slope of the negative peak were computed.

To assess SO/spindle coupling, the SO phase at the spindle peak was estimated using a filter-Hilbert method: the circular mean SO phase (angle) and inter-trial phase clustering (magnitude) metric were used to quantify the consistency of coupling. SO/spindle overlap was also measured as the proportion of spindles overlapping with a detected SO. To account for differences in spindle and SO density, we used randomized surrogate time series to allow for coupling magnitude and overlap metrics as Z-scores, relative to the empirical null distribution. SO phase- spindle frequency modulation was assessed by a circular-linear correlation, with the SO phase split into 18–20-degree bins and instantaneous spindle frequency averaged across bins.

### Spectral power and functional connectivity

Spectral power was estimated using Welch’s method, averaging 0.5 to 20 Hz power spectra across 4-second segments within each 30-second epoch. To assess connectivity between channels, phase slope index (PSI) values were calculated for all channel pairs within 10 minutes of randomly selected N2 sleep epochs, and normalized based on the SD. Only channel-wise net PSI values (the sum of all PSI values for a given channel) are reported here, to reflect whether it was predominantly as a sender or recipient of information from other channels.

### Biological age prediction based on the sleep EEG

We used a modified version of the model described in [27]. This model used 13 features from the sleep EEG and was trained on over 2500 individuals aged 18 to 80 from the Massachusetts General Hospital sleep clinic. The revised model features and weights are available as part of the Luna package (https://zzz.bwh.harvard.edu/luna/ref/predict/). The model uses 13 features from the NREM EEG based on two central mastoid-referenced channels (C3 and C4): mean band power (N3 delta &N1 alpha), spectral kurtosis (N2 delta, theta, alpha, and sigma; N3 theta), time-domain kurtosis (N2 and N3), band power ratios (N3 delta–theta and delta–alpha), *F*_*C*_* *= 13.5 Hz spindle density and number of spindles overlapping a detected SO.

### Statistical analyses: group differences in means and association analyses

In total, 26 metrics were selected for the replication and variability analyses. These metrics describe fundamental properties of hypnogram-derived macro-architecture, spectral power and connectivity during NREM sleep, and NREM transients (spindles & slow oscillations [SO]) and include metrics previously reported to be altered in SCZ [35]. Moreover, the same 26 metrics were reported in our previous publication [7]: here we explicitly attempt direct replication of those prior results. We characterized SS and FS separately given prior evidence for distinct topographies, functional specificity, and SO coupling properties of “FS” and “SS” spindles [25, 36]. We also included PSI as a measure of functional connectivity during N2 sleep due to its insensitivity to volume conduction and additional information on the direction of information flow [37].

To examine if sleep metrics were different between groups or if their association with non-sleep variables was significant, we used linear regression models incorporating age and sex as covariates:


$$\begin{array}{l} Sleep\;\;\;metric\;∼\;Predictor\;\;\;of\;\;\;interest\;+AGE+SEX+error \end{array}$$

where the predictor of interest was either diagnostic status (SCZ vs CTR) or non-sleep variables such as clinical factors, medication, and cognitive scores; for analysis of group difference in means, the sleep metric represented raw estimates derived from the sleep EEG, typically per channel.

To investigate associations between clinical factors, cognitive scores, and medications, we used principal components analysis (PCA). This extracted principal components (PCs) from sets of multi-channel and possibly multi-frequency metrics based on the SCZ group only. For each metric (e.g. spindle density-), the first principal component was selected, except for PSD and PSI, where all components explaining more than 5% of the variance were retained (resulting in 4 and 3 PCs, respectively). For easier interpretability, we flipped the sign of some PCs, such that all PCs had consistent directions in their SCZ-CTR difference (the CTR data were projected onto the SCZ-derived PCs). The selected PCs were tested for association with clinical factors, cognitive scores, and medications. To control for the effects of multiple medications, we performed multiple linear regression, including all drugs simultaneously in the model.

Outlier values (>3 SD from the mean) were removed prior to analysis. We used Glass’s delta to estimate the effect size motivated by its tolerance of the differences in variance between groups [38].

### Accounting for multiple comparisons

To account for comparisons for EEG metrics defined across multiple channels and potentially also multiple frequencies, we utilized a cluster-based permutation using the Freedman-Lane method to correct for nuisance variables [39, 40], as implemented in Luna. We used a clustering heuristic to identify groups of adjacent predictors and tested significance empirically. Adjacency was determined with respect to spatial location (<0.5 Euclidian distance of channels) and, for some metrics, also frequency (<0.5 Hz for PSD, <1 Hz for PSI). Clusters were defined based on an absolute *t*-score threshold *t* = 2. 3000 permutations (based on permuting observed residuals following Freedman-Lane) were used to construct a null distribution to assess statistical significance of the clusters.

### Case–control classification

As previously reported ([7]), a logistic regression model was trained using wave 1 data to classify SCZ and CTR participants based on 12 PCs (Table 2). After projecting all wave 2 individuals into this PC space, we computed the probability of being a case, based on the previous model. Age and sex effects were regressed out before fitting the model. Model performance was evaluated using the area under the receiver operating characteristic curve (AUC).

**Table 2.** Sleep Metrics Tested in Wave 1 ([7]) and Tested for Replication in Wave 2

Domain	# of metrics	Stratifications	# of tests	Multiple comparison correction for replication	# PCs extracted for the prediction model
*Macro-architecture*	*9*	*× 4 stages (for 2 metrics)*	*15*	*None (p-value threshold < 0.01)*	*-*
*Slow oscillations (SO)*	*5*	*× 57 channels*	*285*	*Cluster-based across channels*	*3*
*Spindles (SS & FS as two domains)*	*6*	*× 2 spindle frequencies* *× 57 channels*	*684*	*Cluster-based across channels*	*4*
*Spindle/SO coupling*	*4*	*× 2 spindle frequencies* *× 57 channels*	*456*	*Cluster-based across channels*	*-*
*Channel-wise connectivity (PSI)*	*1*	*× 18 frequencies* *× 57 channels*	*1026*	*Cluster-based across channels & frequencies*	*3*
*Spectral power (PSD)*	*1*	*× 40 frequencies* *× 57 channels*	*2280*	*Cluster-based across channels & frequencies*	*2*

Specific metrics for macro-architecture included time in bed, total sleep time, sleep onset latency, sleep maintenance efficiency, wake after sleep onset time, duration and proportion of N1, N2, N3, R stages, R latency, sleep cycle duration; for SOs—density, duration, negative-peak amplitude, peak-to-peak amplitude, slope; for spindles—density, amplitude, duration, ISA, average frequency, chirp; for Spindle/SO coupling—coupling magnitude, coupling overlap, coupling angle and phase-frequency coupling.

### Inter-participant variability analysis

Group differences in inter-subject variability were tested using Bartlett’s test for homogeneity of variance, with the effects of sex and age regressed out and outlier values (>3 SD from the mean) removed. To estimate the extent to which clinical, cognitive, or medication effects contributed to an increase in between-individual variability in SCZ, we repeated Bartlett’s tests on residuals after accounting for that class of covariate. Specifically, for clinical factors, we controlled for illness duration, total antipsychotics dosage, and 5-factor severity scores; for cognitive factors, we controlled for MST overnight improvement and morning test improvement, MCCB composite and domain scores; for medication effects, we controlled for binary variables indicating the use of a particular type of medication, including medications used by 10 or more patients. For each sleep metric, we first estimated residuals from a linear regression model combining patients and controls with age and sex as predictors, and then further adjusted based on a second Lasso regression model for SCZ individuals only, to remove additional variance associated with clinical, cognitive or medication differences between patients. The optimal lambda penalizing factor was determined separately for each N2 metric using 10-fold cross-validation. The residuals then were compared between groups using Bartlett’s test.

### Dimension reduction

In exploratory analyses, we utilized PCA and uniform manifold approximation and projection (UMAP) [41] as dimensionality reduction techniques applied to selected sets of sleep metrics: those with increased variance in SCZ, or those showing SCZ-CTR mean differences at various significance thresholds (*p-values* <.05, <.01, and <.001). As both methods yielded results lacking visually evident sub-cluster structures, we did not employ further formal clustering methods to attempt cluster identification.

## Results

GRINS wave 2 comprised *N* = 103 SCZ and *N* = 68 CTR individuals, newly collected under the same protocol as wave 1 (see [7] for details). Demographic and sleep variables are given in Table 1, for each wave independently as well as the combined sample (total *N* = 175 SCZ, *N* = 126 CTR). Wave 2 closely resembled wave 1 for primary demographic, clinical, cognitive, and sleep variables. Although wave 2 controls were slightly older with less N3 sleep compared to wave 1 controls (*p-value <* .01) and wave 1 controls were slightly younger (*p-value = *.0104) compared to SCZ individuals, in the combined sample there were no significant case–control differences in either age or N3 duration.

Consistent with wave 1 findings, in the combined sample the SCZ group showed increased time in bed (~1.5 hours, *p-value =* 9 × 10^−12^) and decreased sleep efficiency (effect size [e.s.] = −0.95 SD units, *p-value =* 7 × 10^−6^), primarily driven by longer sleep onset latency. The SCZ group comprised an inpatient sample with a hospital-imposed routine: whereas this implicitly controlled certain factors such as light exposure among patients, it also precluded naturalistic assessment of sleep–wake rhythms and circadian factors. Cases and controls were otherwise well-matched in terms of total sleep time and all stage-specific sleep duration measures (Table 1).

### Replication of sleep alterations reported in wave 1

Following our previous work [7], we computed a battery of metrics mainly focused on the N2 sleep EEG. Specifically, we tested 26 unique classes of the metric across seven key domains (Table 2) to quantify core elements of sleep macro-architecture, fast and SS, SO, spindle/SO coupling, spectral power (PSD), and functional connectivity (PSI summarized channel-wise). Some metrics were calculated for each of the 57 EEG channels and across a range of frequencies, in total yielding 4746 variables. To address multiple testing, in addition to wave 2 providing an independent replication sample, we further used cluster-based permutation to control false positive rates across channels and frequencies, in the initial replication analysis as well as subsequent combined sample analyses. For the latter analyses, we also applied false discovery rate correction within each domain.

**Figure 1.**
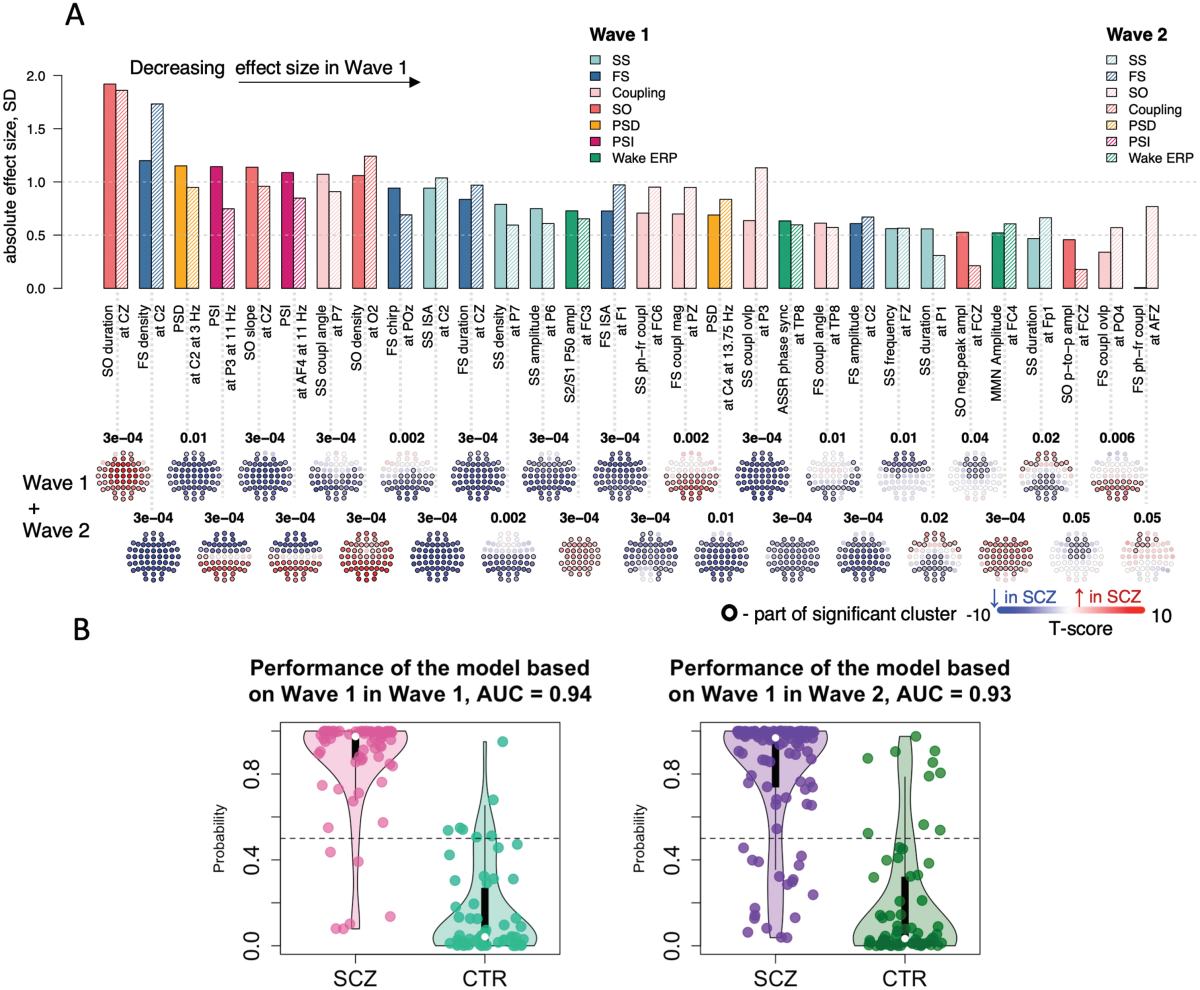
Replication of wave 1 sleep neurophysiology alterations in wave 2 of the GRINS cohort. (A) absolute effect sizes of group-level mean differences in waves 1 and 2, respectively, ordered from highest to the lowest (note: the directions of effect were consistent between waves 1 and 2). Metrics with significant combined sample SCZ-CTR clusters are displayed as topoplots; the EEG channel with the highest absolute t-score was selected for top bar plot. Metrics are ordered from left to right based on decreasing effect size in wave 1. (B) SCZ-CTR classification in wave 2 (Right) using a predictive model derived from wave 1 data (Left).

All previously reported wave 1 sleep EEG group differences replicated in wave 2, based on a significant metric-level result following correction for multiple comparisons and a similar direction of effect (Figure 1A). For example, in wave 1 FS density at C2 was reduced by 28% in SCZ (2.2 vs 3.1 spindles per minute in CTR) and by 30 % (2.3 vs 3.3) in wave 2. Spatial patterns of group differences were broadly congruent between waves, for example, the reduction in SS density in posterior channels (Figure 1A, Supplementary Figure S2).

As expected from the increased sample size, we also detected novel associations in the combined sample which did not pass our stringent significance criterion in wave 1 (cluster statistics are summarized in Supplementary Table S2). These included the SCZ group showing (1) longer frontal and shorter posterior SS duration (two clusters with maximum effects at Fp1 e.s. = 0.55 SD, *p-value =* 7 × 10^−4^ and P1 e.s. = −0.41 SD, *p-value =* .003, respectively), (2) decreased SS frequency in frontal channels (at FZ e.s. = −0.57 SD, *p-value =* 2 × 10^-5^), (3) increased FS/SO coupling magnitude and overlap in posterior channels (at PZ e.s. = 0.86 SD, *p-value =* 5 × 10^−8^ and at PO4 e.s. = 0.51 SD, *p-value =* 8 × 10^−5^ respectively). Only a single metric showed a qualitative difference in the evidence for a statistical association between waves, namely SO-phase FS-frequency modulation, which was increased in frontal channels only in wave 2 (e.g. at AFZ, e.s. = 0.34 SD, *p-value = *.001).

In addition to the replication of individual metrics, we evaluated the performance of our N2-based prediction model to classify diagnostic status. This logistic regression model was trained on wave 1 data only, using 12 PCs summarizing spindle, SO, spectral power, and functional connectivity metrics (see Table 2 and [7] for details). Projecting wave 2 individuals into the predefined 12-dimensional PC space, classification performance in wave 2 was effectively identical to wave 1, with an area under the ROC curve (AUC) value of 0.93 versus 0.94 from the original wave 1 analysis (Figure 1B).

### Spindle density deficits in SCZ and temporal coupling with SO

**Figure 2.**
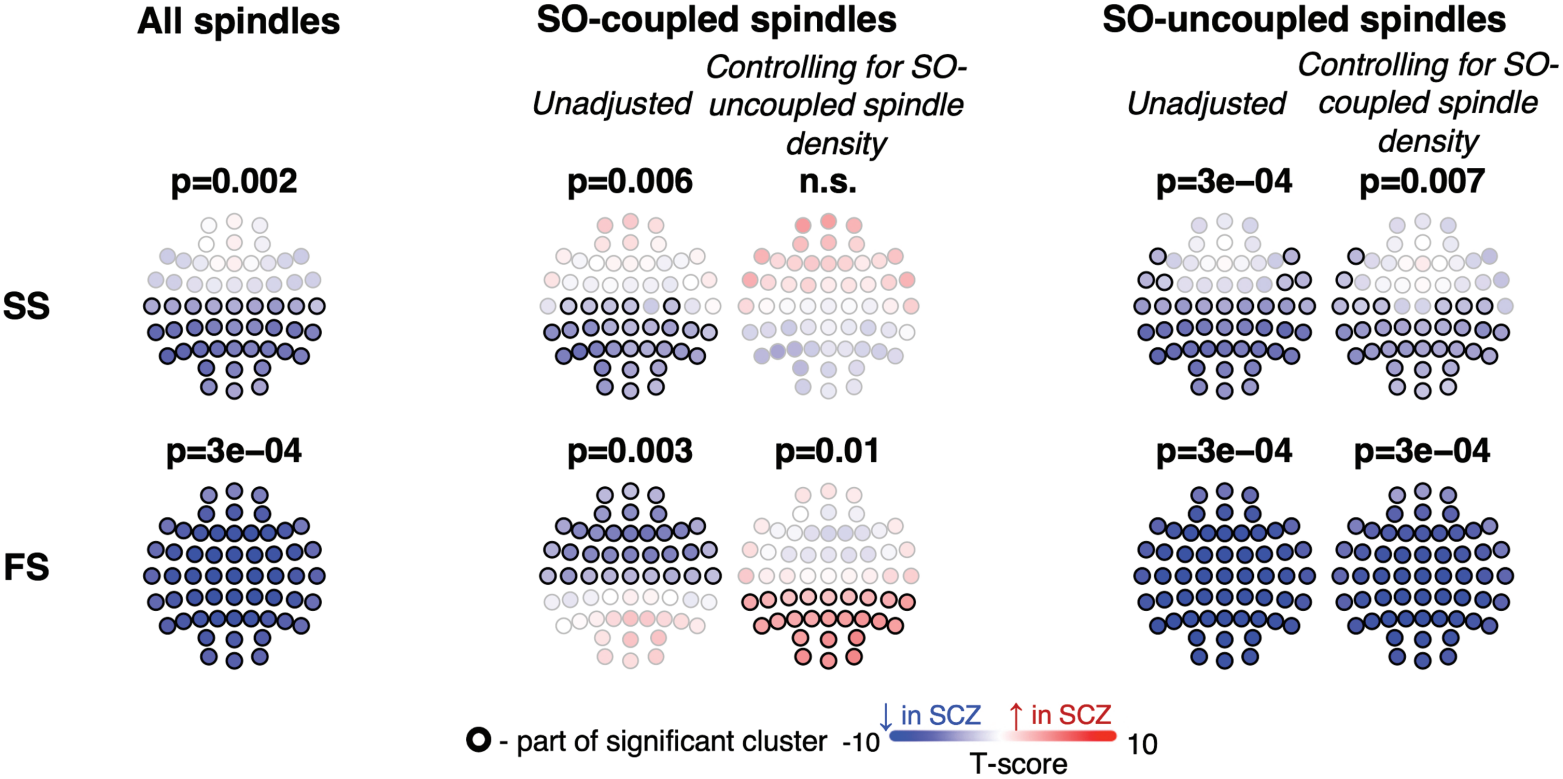
Spindle density deficits in SCZ depend on SO-coupling status. The topoplots represent the group differences between SCZ and CTR in spindle density computed based on all, coupled with SOs and uncoupled with SOs spindles. The first row displays the results for slow spindles (SS), while the second row shows the results for fast spindles (FS).

Having replicated group-level mean differences, subsequent analyses were performed using the combined (*N* = 301) sample. In replication and combined analyses (Figure 1), SCZ patients showed reductions in fast and SS, as well as differences in the rate of and temporal coupling with SO. To further characterize altered spindle/SO coupling in SCZ, we computed the density (count per minute) of SO-coupled and SO-uncoupled spindles separately. Approximately 25% to 45% of spindles (depending on channel and spindle class) overlapped (“coupled” with) a detected SO (Supplementary Figure S3). Case–control reductions in overall spindle density reflected qualitatively different effects for coupled and uncoupled spindles (Figure 2). For both fast and SS, the overall reductions in spindle densities were largely driven by fewer SO-uncoupled spindles in patients. In contrast, controlling for SO-uncoupled spindle density, SO-coupled spindles either showed no group differences (SS) or even a significant increase in patients (fast occipital spindles). This latter result is consistent with the previous significant increase seen for the fast spindle-SO overlap statistic: in patients there are fewer fast spindles (FS) overall, although some types (specifically, fast parietal and occipital SO-coupled spindles) are relatively over-represented, underscoring that topographical and temporal contexts are important to consider when evaluating spindle activity.

### Greater between-individual variability among patients with SCZ across diverse sleep metrics

**Figure 3.**
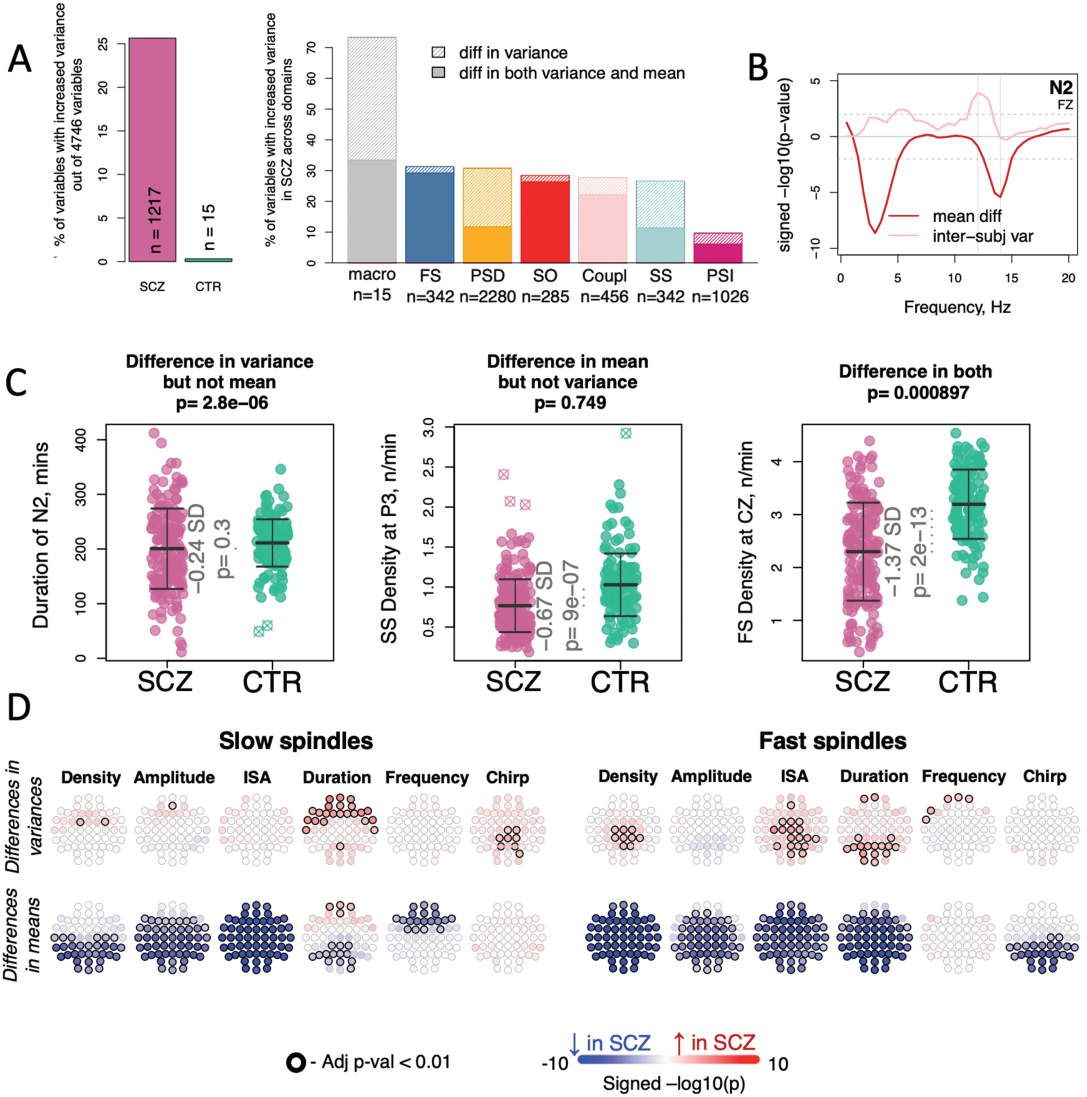
Increased variability in the SCZ group across multiple sleep estimates. (A) Left: the percentage of sleep variables with significantly (*p*-value < .05) increased variability in SCZ vs CTR (magenta bar) and increased variability in CTR vs SCZ (green bar); Right: the percentage of sleep variables with increased variability (light shade) and those with both increased variability and altered means (dark shade) across seven domains. (B) visualization of differences in means and interindividual variances of spectral power at FZ across frequencies during the N2 stage; vertical lines represent 12 and 14 Hz. (C) examples of sleep variables with the difference in variance (Bartlett’s test *p*-value in the title with effects of age and sex regressed out prior statistical comparison), but not in means (effect size and *p*-value from linear regression controlling for age and sex inside the graph); in mean, but not in variance; and in both mean and variance. (D) topoplots illustrate the distinct profiles of between-group differences in variance (top row) versus mean (bottom row) all channels for FS and SS metrics. Significant channels after the FDR adjustment for multiple comparisons (*N* of tests = 1368—differences in variance and means for SS and FS across 6 metrics across 57 channels—2 × 2 × 6 × 57) highlighted with a black rim.

We next focused on between-individual variability for the sleep metrics in Table 2. Of these, 1232 (26%) exhibited nominally (*p-value <* .05) significant differences, based on Bartlett’s test comparing the between-individual variances within the SCZ group versus within the CTR group, after removing outliers and adjusting for the effects of age and sex. Of note, all but 15 significant tests pointed to higher variability in the SCZ group (Figure 3A).

Macro-architecture metrics expressed some of the largest variance increases in SCZ. For example, whereas N2 duration showed no significant difference in means (201 vs 209 minutes, *p-value =* .26), cases had a markedly higher SD of 71, versus 47 for controls (Bartlett *p-value =* 3 × 10^−6^). Total sleep time, N3 and REM duration likewise showed similar increases in variability in SCZ. This signature of increased SCZ variability was further observed across all domains of N2 micro-architecture. Whereas for some domains, metrics exhibiting increased variance among SCZ individuals almost always showed concurrent significant mean differences (primarily FS, SO, and spindle/SO coupling), metrics in other domains also exhibited variance differences in the absence of corresponding group difference in means (primarily macro-architecture, SS and PSD domains, Figure 3A).

Figure 3B shows SCZ-CTR differences for stage N2 spectral power at a representative frontal channel (Fz): whereas slower sigma frequencies (~11.5 Hz) showed different variances (higher in SCZ) but equivalent means, faster sigma frequencies (13–16 Hz) showed the opposite pattern, of similar variances but a significantly lower mean in SCZ. This pattern was generally more pronounced in anterior channels. As a second illustration of these divergent effects, Figure 3C shows three exemplar metrics with qualitatively distinct alterations in SCZ: in variance only (N2 duration), in means only (posterior SS density), or in both (central FS density). Similarly, primary spindle metrics showed distinct patterns for group differences in variances versus means (Figure 3D).

In principle, increased interindividual variability in SCZ is consistent with the hypothesis that the sleep EEG stratifies patients into discrete subtypes. However, we did not find evidence of distinct patient-specific clusters readily emerging from exploratory analyses using either PCA or UMAP dimension reductions, whether based on metrics showing increased variances or with altered means in the SCZ group (Supplementary Figure S4).

### Patient heterogeneity in clinical and cognitive factors

**Figure 4.**
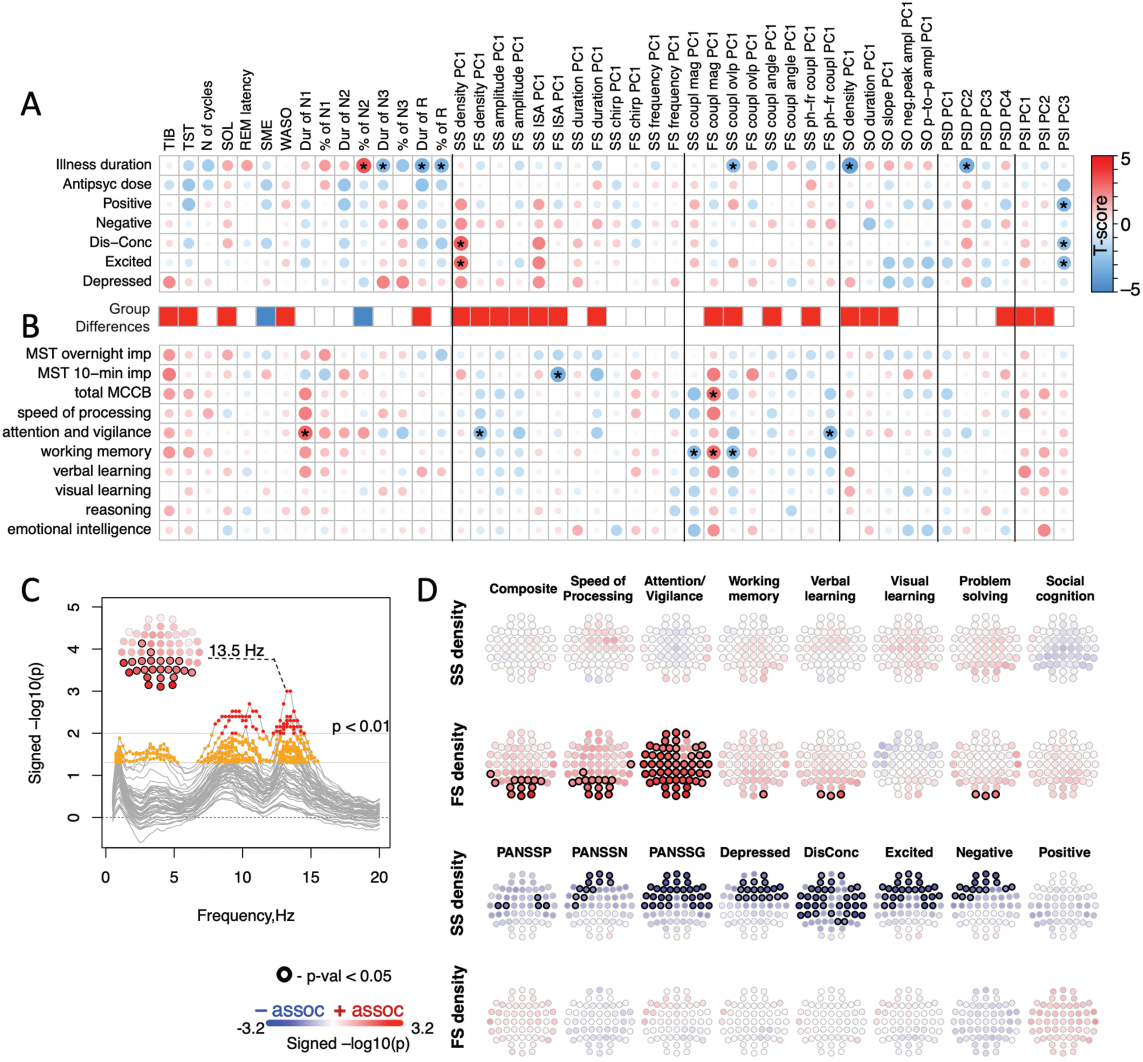
Sleep EEG associations with clinical and cognitive factors. A—the matrix shows t-scores from linear regressions (controlling for age and sex) between sleep features and clinical factors in SCZ. For multi-channel variables, we used the first PC (or all PCs explaining > 5% of total variance across channels × frequencies in case of PSD and PSI) derived separately for each metric and aligned all PCs by the sign of the difference between SCZ and CTR means (i.e. positive value means that clinical factor was associated with metric exhibiting more SCZ-like pattern and negative—CTR-like pattern). Stars mark associations with *p*-value < .01. The horizontal bar below the matrix illustrates SCZ-CTR differences in the corresponding variable: blue = decrease, red = increase, white = no difference; (B) same as A but for the cognitive (instead of clinical) variables. (C) the graph illustrates the association between the total MCCB score and spectral power across all channels (lines) in the frequency range of 0.5–20 Hz. Channels and frequencies displaying association above the nominal significance threshold of *p*-value < .05 are highlighted in orange and with *p*-value < .01 in red. (D) topoplots represent channels where the association between spindle density and total MCCB score (top two rows) or PANSS scores (bottom two rows) were nominally significant (*p*-value < .05).

We next asked whether the increased N2 heterogeneity among SCZ patients may be reflecting patient-to-patient differences in illness duration, symptoms, or cognitive deficits, given that these factors did exhibit associations with some sleep metrics (Figure 4, A and B). We created new sets of sleep metrics for patients, that adjusted for either clinical (illness duration, total antipsychotics dosage, and PANSS scores) or cognitive variables (MST overnight improvement and morning test improvement, MCCB scores), using residuals from patient-only Lasso regressions that fit each sleep metric jointly on all clinical or cognitive variables, thereby removing the between-patient variability explained by these factors. Repeating the Bartlett tests, the SCZ group nonetheless continued to exhibit significantly higher variabilities for the majority of the previously reported 1217 metrics: either 1110 or 1139, controlling for clinical or cognitive variables, respectively (Supplementary Figures S5A).

The persistence of increased N2 variability in SCZ suggests that objective sleep biomarkers may capture disease-relevant factors that are not otherwise well-characterized by existing clinical and cognitive measures. Nonetheless, it does not imply that N2 sleep metrics were unrelated to clinical and cognitive factors in patients (Figure 4, A and B). Although a comprehensive examination is beyond the scope of this manuscript, we performed a series of secondary analyses focused on patient MCCB and PANSS scores in relation to N2 spectral power and spindle density. Higher MCCB composite scores were associated with increased occipital and parietal N2 sigma-range power (maximal at 13.5 Hz, *p-value <* .01, Figure 4C). Consistent with this, MCCB composite scores were also associated with increased fast spindle density (Figure 4D). Considering the separate MCCB domains, this effect was most pronounced for Attention/Vigilance and Speed of Processing domains and was restricted to FS. In contrast, clinical measures (three primary PANSS scores and derived five-factor scores [42]) showed nominal (*p-value < *.05) associations with slow spindle densities at multiple central and frontal channels, across most clinical measures, whereby reduced slow spindle activity predicted more severe symptoms (Figure 4D).

### N2 predictors of sleep-dependent motor procedural memory in SCZ

Sleep-dependent MST performance improvement has previously been associated with greater %N2 in healthy individuals and fast sleep spindle density in SCZ patients [43–46]. We observed a robust attenuation of overnight improvement of MST performance in the SCZ group versus controls (percentage, e.s. = −0.99, *p-value* = 4 × 10^−9^, Supplementary Figure S6) while learning rate during training was not different between the groups (*p-value >* .05), consistent with prior reports [46–48]. However, neither %N2 nor sleep spindle density was associated with overnight MST improvement in the SCZ group (Figure 4B).

### Medication effects

**Figure 5.**
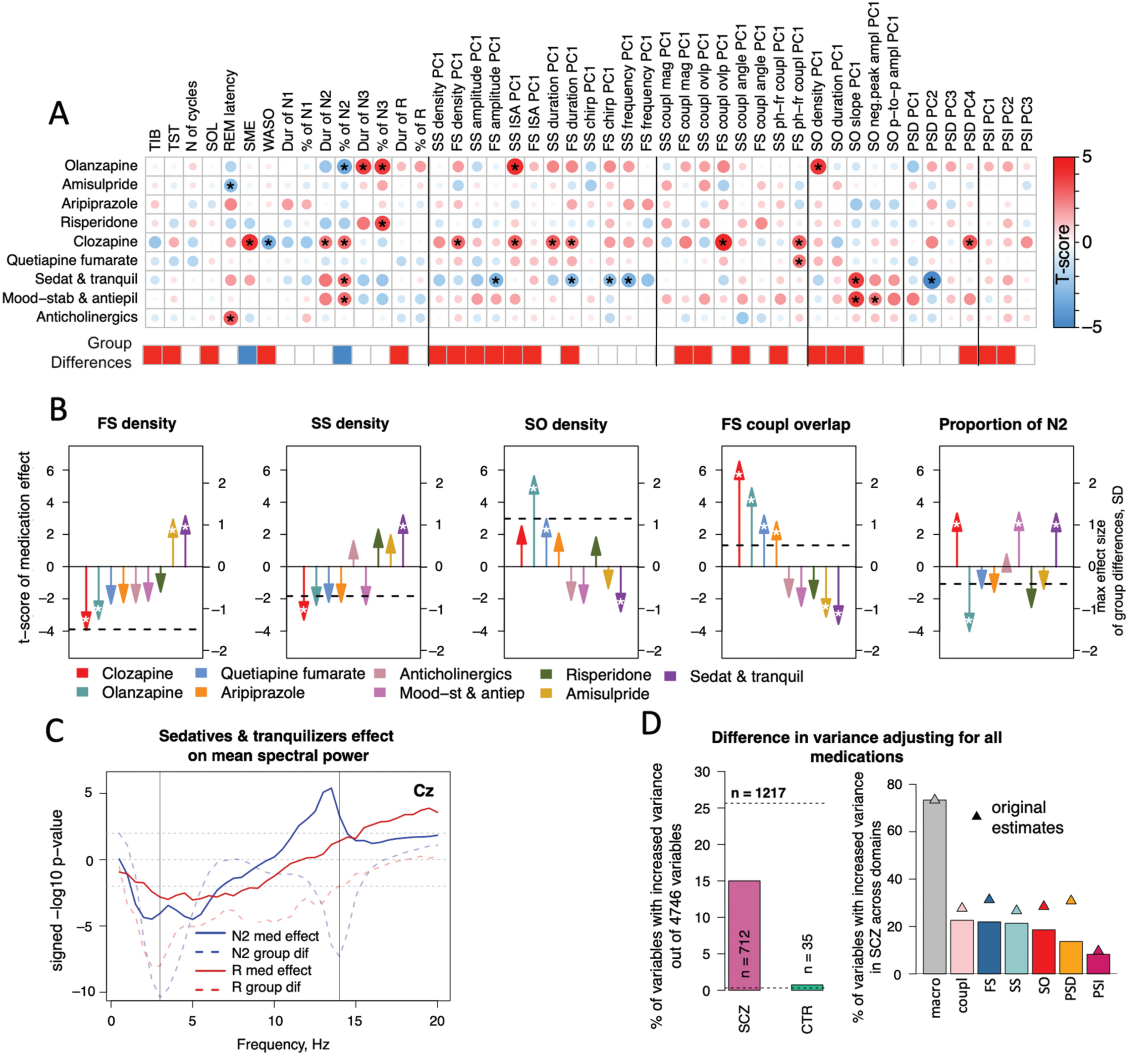
Sleep EEG associations with medication use in SCZ. A—the matrix shows t-scores from a multiple linear regression where all medication groups were included as well as age, sex and illness duration. between sleep features and binarized medication use in SCZ (where each medication is included separately). For multi-channel variables, we used the first PC (or all PCs explaining >5% of total variance across channels × frequencies in case of PSD and PSI) derived separately for each metric and aligned all PCs by the sign of the difference between SCZ and CTR means (i.e. positive value means that clinical factor was associated with metric exhibiting more SCZ-like pattern and negative—CTR-like pattern). Stars mark associations with *p*-value < .01. The horizontal bar below the matrix illustrates SCZ-CTR differences in the corresponding variable: blue = decrease, red = increase, white = no difference; (B) examples of medication effects on FS density, SS density, SO density, FS coupling magnitude and N2 proportion. Each arrow indicates an effect (t-score) of a certain medication on a sleep variable from a multiple linear regression where all medication groups were included as well as age, sex and illness duration. Nominal significance (*p*-value < .05) is marked by a white star inside the arrows. For multi-channel metrics, the largest effect across all channels is presented. The horizontal dashed line indicates the effect size of the group difference between SCZ and CTR in the corresponding metric (at the channel with the largest effect size for multi-channel variables). (C) sedatives and tranquilizers effect on spectral power in N2 and REM across frequencies (solid lines) in comparison to SCZ-CTR differences (dashed lines) at Cz channel (D) left bar plot shows the percentage of all sleep variables still with higher variance in SCZ or CTR group after effects of all common medications have been simultaneously regressed out using LASSO regression for SCZ group, compared to the original estimates (horizontal dashed lines); the right bar plot is similar but stratified by domain (denominator for percentages is the number of variables in the domain); original estimates are marked by triangles.

Medication use showed an array of robust effects on both macro- and micro-architecture sleep metrics (Figures 5, Supplementary Figure S7). Each patient’s medication use at the time of the sleep study was encoded as a binary vector of nine medications (six antipsychotics and three categories of adjunct drugs, each used by at least 10 patients) and entered into a case-only linear model to predict each sleep metric, controlling for age and sex. Among antipsychotic medications, olanzapine (*N* = 81 patients) and clozapine (*N* = 22) affected sleep metrics the most (Figures 5, A and B), albeit sometimes in qualitatively different ways. For example, whereas olanzapine use was associated with decreased N2 proportion (by −10 % compared to patients not taking olanzapine), clozapine use had the opposite pattern of increased N2 (+ 14%, Figure 5B right). Clozapine use was also associated with decreased FS density, replicating a prior report [46].

Among adjunct medications, sedative, and tranquilizer use (*N* = 45) had the most marked effects on sleep micro-architecture, impacting multiple spindles (e.g. increased FS duration, *p-value =* 4 × 10^−4^, Supplementary Figure S7) and SO characteristics (e.g. altered slopes, *p-value =* 3 × 10^−4^). Sedative and tranquilizer use was also associated with decreased 1–7 Hz power during N2 (cluster *p-value =* .009, Figure 5C). Additionally, sedatives and tranquilizers were linked to increased power in the sigma frequency range. To test whether such effects were specific to N2 sleep we compared them to findings during R: while the association in 1–7 Hz power was also present during R, albeit with an attenuated effect, the sigma association was specific to N2 sleep.

Given the variability in patterns of medication use, combined with the marked and sometimes divergent associations with the sleep EEG, we posited that medication use would account for some of the increased variability in sleep metrics found in SCZ. Adopting the same approach as above (adjusting for medication use in patients prior to testing for group differences in variance using Bartlett’s test), we observed a greater—but still only partial—drop in the number of metrics showing increased variance in SCZ. The largest declines were in the domain of spectral power, from approximately 30% to 15% of all metrics that were significantly more variable, compared to the expected rate of 5% given the nominal significance threshold of *p-value =* .05 (Figure 5D). Nonetheless, across all domains, 712 metrics—a rate far greater than expected by chance—still showed greater variance among SCZ patients, compared to only 35 metrics with a higher variance in controls. The increase from 15 to 35 variables with significantly higher variance in the CTR group after we accounted for medication in the SCZ group was due to reduced variance in patients (i.e. vs controls).

### Accelerated age-related NREM alterations in SCZ

**Figure 6.**
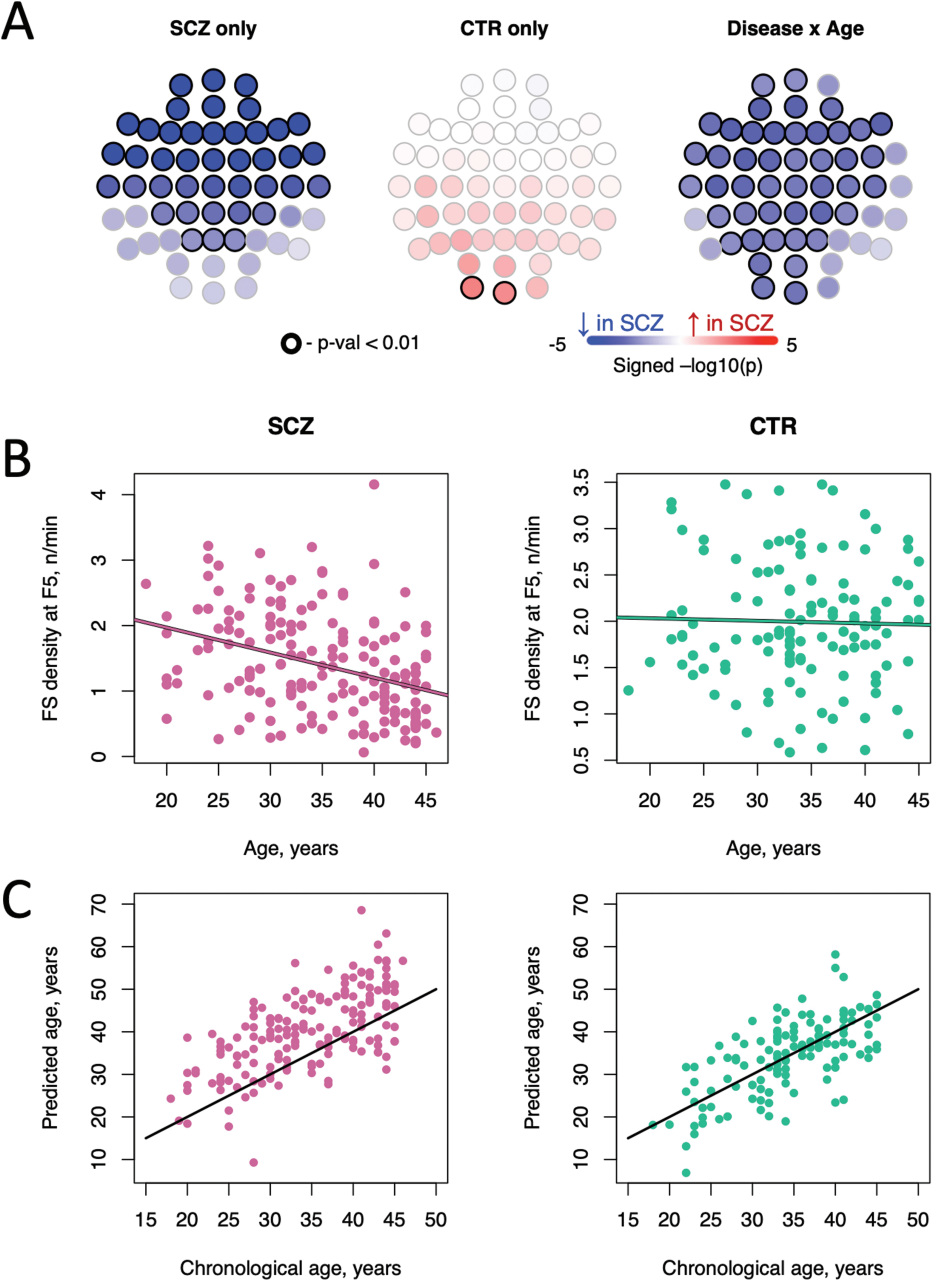
Fast spindle density shows differential age-related decline in SCZ. (A) the topoplots show the associations between FS density and age, in either a case-only (left plot) or control-only (middle plot) analysis, and the interaction *p*-values from the joint models (right plot); associations *p*-value < .01 are marked by dark circles; (B) scatter plots showing FS density at F5 as a function of age separately in cases and controls. (C) scatter plots showing predicted and observed ages separately in cases and controls; age prediction was based on a modified version of the model described by [27]).

Finally, we asked whether SCZ patients had greater sensitivity to other factors known to influence sleep in the general population—in particular, age and sex (note: these were statistically controlled in the above analyses). In exploratory models including interaction terms allowing the effects of age and sex to vary between cases and controls, the most prominent interaction involved age and FS density across multiple channels, such that the SCZ group showed greater age-related declines (maximal effect at F5, interaction *p-value =* 4 × 10^−4^; 46 channels had an interaction *p-value <* .01, Figure 6A). This effect was specific to FS: SS density interaction terms were nonsignificant (*p-value >* .05) for all channels. Sex did not appear to modify case–control differences in either FS or SS (all *p-value >* .05). Although spindle density decreases in older adults [25], in our middle-aged (median age 34, IQR 30.5–39) sample FS density was largely independent of age in controls (e.g. *r* = −.03, *p-value =* .76 at F5). In contrast, the SCZ group showed a pronounced age-related reduction in FS density (*r* = −.35, *p-value =* 4 × 10^−6^), consistent with an accelerated aging effect among individuals with SCZ (Figure 6A).

Key macro-architecture metrics including TST also showed significant age × disease interactions. Although patients were well-matched to controls in terms of age (*t*-test and Bartlett test *p-value =* .68 and *p-value =* .19, comparing means and variances in age between groups, respectively), patients showed a highly significant age-related reduction in TST (*r* = −.32, *p-value =* 1 × 10^−5^), whereas TST and age were unrelated in controls (*p-value =* .57), yielding a significant age × disease interaction (*p-value =* .003). Controlling for illness duration and age-at-onset did not alter the TST-age association in patients and neither term was associated with TST conditional on age. Likewise, patient FS density was not associated with either illness duration or age-at-onset when controlling for chronological age.

To more directly address the question of accelerated aging, we used an independently developed model to predict so-called brain age from the sleep EEG ( [27], see Methods for details). Predicted and chronological ages were similarly correlated in patients (*r* = .65) and controls (*r* = .67). However, predictions for patients were on average 5.8 years greater than their chronological age (*p-value <* 10^−15^ one-sample *t-*test), whereas in controls predicted and chronological ages had similar means (−0.1 year difference, *p-value =* .85). Consequently, the predicted age difference (PAD = predicted age—chronological age) was significantly higher in patients compared to controls (*p-value =* 10^−12^, also covarying for chronological age and sex), consistent with accelerated aging.

In patients, PAD was not associated with duration of illness (*p-value =* .19 in a model controlling for chronological age and sex) and, similar to the full SCZ group, earlier-course patients (≤5 years from diagnosis, *n* = 43) displayed increased PAD by 6.6 years compared to CTR. In general, the earlier-course subset showed similar differences in means and variances (vs CTR) compared to the overall patient group (data not shown). PAD scores also did not show greater variability among SCZ patients compared to controls (Bartlett *p-value =* .27). In controls, the mean PAD varied between males (+1.17 years) and females (−1.87 years) significantly (*p-value =* .007). In contrast, the PAD scores in male (+6.4 years) and female (+5.0 years) patients were not significantly different from each other (*p-value =* .24).

PAD scores were not associated with clinical (PANSS) or cognitive (MCCB) scores (all *p-values >* .05), although they were associated with medication use. Most notably, patients using olanzapine (*N* = 81) had significantly (*p-value =* 2 × 10^−8^) older PAD scores (+8.98 years) compared to patients not using olanzapine (*N* = 94; mean + 3.08 years). This effect remained significant (*p-value = *3 × 10^−6^) when jointly controlling for other medications used; in this joint, case-only model, clozapine, anticholinergics, and emotion stabilizers/antiepileptics were also associated with significantly older PAD scores (Supplementary Table 3). These effects were not driven by only one or two model features: for example, similar to the original SCZ-CTR contrast, olanzapine use was significantly associated with the majority of the model’s features (Supplementary Table S4). Consistent with the lack of association between PAD and MCCB scores, we did not observe any significant associations in cases between cognitive scores and medication use (all *p-values* ≥ .05, e.g. olanzapine-MCCB total score *p-value* = .81).

Importantly, core NREM deficits persisted even in the minority (*N* = 44, 25%) of patients not taking any of the medications associated with higher PAD scores (namely olanzapine, clozapine, anticholinergics, emotion stabilizers or antiepileptics). In particular, whereas this selected subgroup had PAD scores that were not significantly different from CTRs (*p-value* = .3), we nonetheless observed significantly reduced fast spindle densities across all 57 channels versus CTRs (all *p-value* < .01, minimum *p-value* = 5 × 10^−6^) and the extent of this deficit was similar to that seen in the majority of patients taking at least one of these medications (case-only *p-values* > .05 for all 57 channels, comparing the *N* = 44 subgroup to the remaining *N* = 175–44 cases).

## Discussion

In a large high-density EEG study of sleep and SCZ, we found unambiguous support for our previously reported alterations of sleep neurophysiology [7], observing comparable effect sizes and directions. Our predictive model of diagnostic status, previously trained on only the first wave of data, rendered effectively identical classification accuracy in the independent second wave. Overall, these replicated results implicate not only reduced fast spindle density as a core feature of SCZ but also multiple aspects of N2 sleep including SS, spindle morphology, SO features, and spindle-SO coupling, as well as differences in spectral power and patterns of functional connectivity. Our results also support the relevance of the distinction between fast and SS: for example, in patients, we observed qualitatively different patterns of results for these spindle classes with respect to patterns of age-related decline in spindle density, as well as differential associations with cognitive versus clinical features. We further identified instances where the topographic and temporal context of spindles played a critical role, including the relative increase in SO-coupled posterior FS in patients, potentially reflecting the potentiating effect of SO state on spindle generation, which may become a more critical factor in individuals with otherwise disrupted spindle-generating circuits.

Independent of group differences in means, patients with SCZ showed significantly increased person-to-person variability for many sleep metrics considered. This echoes previous reports of higher between-individual variability in brain morphology [49, 50] and functional connectivity [51] in SCZ, as well as the long-standing nosological discussion of heterogeneity [52, 53]. Such heterogeneity could be linked to the existence of quasi-discrete SCZ subtypes—a hypothesis tested by prior studies attempting to derive subtypes based on symptom profiles, the presence of affective symptoms, or cognitive functioning, but which failed to show clear links to specific neurophysiological mechanisms [54]. Research examining SCZ and schizoaffective disorder reported that both disorders shared key cognitive, social cognitive, and neural properties and were indistinguishable by those factors [55]. Although not a primary focus of this study, preliminary analyses did not suggest clearly distinct patient subgroups on the basis of N2 alterations. Rather, our results point to a continua or spectrum of N2 alterations among patients. This spectrum of N2 changes may reflect impairments in multiple neurocircuits, consistent with the polygenic nature of SCZ disease risk that suggests many biological pathways underlying its pathophysiology.

Whereas cognitive deficits are a feature of SCZ [11, 56], multiple clinical and cognitive factors exhibit significant diversity among patients [57, 58]. Reduced sleep spindles in SCZ have been suggested to represent a treatable endophenotype linking SCZ risk genes to impaired cognition [59]. Increased FS density predicted fewer cognitive deficits (in particular, MCCB attention/vigilance scores) and increased SS density predicted less severe symptoms. However, these associations were generally modest, and the observed variability in clinical and cognitive factors could not account for the increased patient variability in sleep.

With regard to MST performance, although we replicated prior findings of impaired sleep-dependent memory consolidation in SCZ [60, 61], previous reports linking overnight MST improvement and spindle density [43, 46] were not supported by the current study. Future work will be needed to resolve these apparent discrepancies, whether they reflect purely statistical (type I or II) errors, or systematic differences in factors such as sample composition, demographics, medication regimens, or inpatient versus outpatient settings.

Patterns of medication use induced significant heterogeneity in patient sleep EEG metrics. Although several relatively small studies reported direct, typically acute effects of antipsychotic use on sleep [62–65], our study focused on a naturalistic setting of chronic use in a wide range of antipsychotics, with patients often on multiple medications concurrently. Nonetheless, consistent with our findings, previous studies have shown acute olanzapine use causes reduced spindle activity in both healthy controls [66] and patients [63], and also identified different effects of individual antipsychotic drugs on spindle activity [14]. Even though all antipsychotics considered here belonged to the second-generation (or atypical) class of pharmaceuticals, their associations with the sleep EEG were diverse. All antipsychotics share the mechanisms of inhibiting dopamine D2 receptors, while displaying different pharmacology across D1, D3, D4, and D5 receptors, as well as on serotonin receptors, adrenergic receptors, M1, and H1 receptors [67]. Such polypharmacy on multiple G protein-coupled receptors of antipsychotics potentially underlies their effects on neurophysiology as some of these receptors play a role in sleep regulation [68, 69]. For example, animals with a loss of function of the serotonin receptor gene 5-HT1A showed increased NREM duration [70]. Clozapine is recognized as a potent norepinephrine ɑ-2 receptor antagonist and a norepinephrine reuptake inhibitor [71] and could influence sleep architecture in patients with schizophrenia due to the involvement of the noradrenergic system in regulating the sleep–wake cycle [72]. More generally, our results underscore the importance of more granular control for medication effects in future studies: one of the most common approaches—using the total antipsychotic dosage equivalent to chlorpromazine—lacked significant associations with key sleep metrics, despite clear effects of individual medications. Other medication classes including sedatives, commonly used as adjunct medications in SCZ, had large effects on sleep macro and micro-architecture, as others have noted [14]. Sedatives increased both slow and fast spindle density, consistent with the premise of prior literature that examined their role in treating memory deficits in SCZ patients [43, 73].

Adjusting for medication use accounted for a substantial proportion of the heterogeneity in sleep metrics, based on the number of metrics with significantly increased variance in SCZ. The interpretation of associations with medication use in the sleep EEG is nonetheless challenging: a given effect could be (1) a purely epiphenomenal side-effect, (2) a marker of therapeutic action, normalizing canonical deficits, or (3) a consequence of nonrandom prescription of particular medications to particular patients, based on clinical course. That is, associations between certain medications and the sleep EEG may not always be simple confounds per se, but instead reflect an individual’s particular deficits, an allostatic response to those deficits, or a personalized response to treatment, as well as the specific properties of the particular treatment on other molecular targets.

Adjustment for medication effects still left a significant portion of the between-patient variability in N2 sleep unexplained; however, which presumably reflects genetic and environmental risks as well as developmental and dynamic sources of intraindividual variability, although there are mixed findings regarding the role of genetics in driving between-patient variability. For example, whereas one study found no relation between genetic risk of SCZ and brain structural heterogeneity [49], another found a significant association between SCZ polygenic risk and a greater number of brain regions displaying deviations in cortical thickness [74].

One possible driver of higher between-individual variability in SCZ is greater reactivity to other exogeneous factors that impact sleep in the general population, including demographics. Indeed, patients tended to exhibit greater age-related changes for some key metrics (fast spindle density, total sleep time). Such patterns of greater age-related changes, however, were not observed for all metrics displaying comparably large case–control differences. Based on an independent prediction model of brain age from the sleep EEG, patients showed profiles of NREM sleep metrics consistent with accelerated aging. Although the extent to which biological age as predicted from the sleep EEG captures the same phenomena as age predicted from MRI imaging (or other modalities including epigenetics) is unclear, our findings were consistent with a recent meta-analysis that found increased brain ages in patients compared to controls, but no relationship with clinical factors in patients [22].

Also consistent with [22], there was no association between PAD and CPZ equivalent dose, although we did find a large effect of olanzapine use, with use being associated with advanced aging, and similar effects for clozapine, anticholinergics, and emotion stabilizers/antiepileptics. These findings echo a growing literature suggesting that antipsychotics can have adverse cognitive effects driven by their anticholinergic properties [16–18]. In our sample, both olanzapine and clozapine (which have relatively high anticholinergic burdens) were associated with accelerated PADs, as was the use of anticholinergics, whereas amisulpride, risperidone, and aripiprazole (that have lower anticholinergic burdens) were not. Our study did not find direct associations between medication use and cognition. However, given the existing literature connecting NREM physiology and cognitive functioning [75, 76], it is intriguing to hypothesize that the sleep EEG constitutes a sensitive precursor of cognitive alterations that may arise from the progression of disease and/or the detrimental effects of chronic antipsychotics use, analogous to biomarkers such as tau burden tracking early stages of Alzheimer’s disease years before symptoms emerge [77]. Olanzapine has been shown to impact brain morphology, including reduced cortical thickness in a placebo-controlled randomized clinical trial [78], and these or other effects may be manifest in our EEG-based finding of accelerated aging. As such, medication effects on the EEG may not solely reflect state-dependent and reversible confounding, but instead reveal true physiologic differences in the brains of patients, induced chronically through medication side effects. Given that the sleep EEG is more directly transferable to animal-based preclinical studies than cognitive testing or MRI brain imaging, it may prove to be a critical assay for studies aiming to develop new antipsychotics with lessened adverse effects on cognition. Studies might also investigate the clinical potential for EEG-based predictors of future adverse cognitive side effects for specific individuals and medications.

Our study is not without caveats and limitations. Of note, the inpatient context likely impacts the extent to which circadian factors and daily rest–activity rhythms play a role. Although we attempted to document and control medication effects among patients, it is still challenging to fully account for such effects (including chronicity of medication use) in the absence of unmedicated patients. While we investigated the effects of multiple medications in relatively large subgroups, medications used by only a handful of patients were excluded from analyses. Additionally, examination of acute intraindividual medication effects on sleep parameters in the same group of patients was not possible due to our cross-sectional design, which focused on patients with largely stable medication regimens. Translational research using animal models may help address the impact of acute or chronic administration of these antipsychotics and adjunct medications. In addition, characteristics of drug naïve patients and longitudinal studies may provide evidence of sleep EEG metrics changes before and after medication use. Finally, although previous studies reported night-to-night stability in SCZ with respect to spindle deficits over short timescales [36, 79], our restriction to a single night still limits our ability to disentangle within-patient night-to-night variability (over timescales from days to years) from more “trait-like” between-patient factors.

In summary, the current study offers substantial evidence of robust and replicable alterations in sleep neurophysiology as well as increased variability among patients with SCZ. Part of this increased variability may be explained by an acceleration of normal age-related changes in patients. Part—but not all—may be explained by patterns of medication use, which will be important to more directly model and disentangle in future studies aiming to more precisely link clinical and cognitive outcomes to sleep physiology. Group-level mean differences in NREM neurophysiology have now been unambiguously established, although the extent to which they aggregate both causal and noncausal factors remains to be resolved. The substantial heterogeneity in sleep architecture among patients, as well as their cognitive and clinical symptoms, points to the need for large, transdiagnostic, demographically diverse, genotypically informative, and deeply phenotyped samples to characterize the underlying links between sleep and individual patient characteristics. We speculate that sleep neurophysiology may offer a unique window on the etiological and genetic diversity that underlies SCZ risk as well as treatment response and prognosis. Future efforts should be aimed at elucidating how sleep neurophysiology changes in the high-risk group, and their connection to genetic factors, as well as a better understanding of when differences in sleep emerge and their progression over the course of the disorder. Longitudinal and other approaches will be necessary to determine whether this increased interindividual variability is also reflected in altered intraindividual variability, that is, the temporal stability of the sleep EEG over different timescales, from seconds to days to years. Finally, as well as potentially predicting future adverse cognitive side-effects, our findings related to medication suggest that sleep parameters could serve as pharmacological target engagement markers, enhancing our understanding of treatment response in SCZ.

## Supplementary material

Supplementary material is available at *SLEEP* online.

zsae218_suppl_Supplementary_Figures_S1-S7_Tables_S1-S4

## Data Availability

Anonymized individual-level data for the derived EEG metrics and the code to generate the key figures are available from the corresponding author upon reasonable request.
