# Supplementary material for: A spectrum of altered non-rapid eye movement sleep in schizophrenia
Source: Sleep. 2024 Sep 19;48(2):zsae218. doi: 10.1093/sleep/zsae218 (PMC11807884; doi:10.1093/sleep/zsae218)
Supplement: zsae218_suppl_Supplementary_Figures_S1-S7_Tables_S1-S4 [file zsae218_suppl_supplementary_figures_s1-s7_tables_s1-s4.docx]

**SUPPLEMENTAL MATERIAL**

**A spectrum of altered non-rapid eye movement sleep in schizophrenia**

**Authors:** Nataliia Kozhemiako^1†^, Chenguang Jiang^2†^, Yifan Sun^2^, Zhenglin Guo^3^, Sinéad Chapman^3^, Guanchen Gai^2^, Zhe Wang^2^, Lin Zhou^3^, Shen Li^4^, Robert G. Law^1^, Lei A. Wang^3^, Dimitrios Mylonas^5^, Lu Shen^7^, Michael Murphy^4^, Shengying Qin^7^, Wei Zhu^2^, Zhenhe Zhou^2^, Robert Stickgold^9,10^, Hailiang Huang^3,8^, Shuping Tan^6^, Dara S. Manoach^5^, Jun Wang^2•^, Mei-Hua Hall^4•^, Jen Q. Pan^3•*^ & Shaun M. Purcell^1,10•*^

**Affiliations:**

1. Department of Psychiatry, Brigham and Women’s Hospital, Harvard Medical School; Boston, USA
2. The Affiliated Wuxi Mental Health Center of Nanjing Medical University; Wuxi, China
3. Stanley Center for Psychiatric Research, Broad Institute of MIT and Harvard; Boston, USA
4. Department of Psychiatry, McLean Hospital, Harvard Medical School; Boston, USA
5. Department of Psychiatry, Massachusetts General Hospital, Harvard Medical School; Boston, USA
6. Huilong Guan Hospital, Beijing University; Beijing China
7. Bio-X Institutes, Shanghai Jiao Tong University; Shanghai China
8. ATGU, MGH, Harvard Medical School; Boston, USA
9. Beth Israel Deaconess Medical Center; Boston, USA
10. Department of Psychiatry, Harvard Medical School; Boston, USA

^†^ - co-first authors; • - co-senior authors

* - corresponding authors (Jen Q. Pan, jpan@broadinstitute.org ; Shaun M. Purcell, smpurcell@bwh.harvard.edu)

| AASM | American Academy of Sleep Medicine |
| --- | --- |
| AUC | Area under the ROC curve |
| CPZ | Chlorpromazine |
| CTR | Controls |
| DSM-5 | Diagnostic and Statistical Manual of Mental Disorders, Fifth Edition |
| e.s. | Effect size |
| EEG | Electroencephalography |
| ERP | Event-related potential |
| FDR | False discovery rate |
| FS | Fast spindle |
| GRINS | Global Research Initiative of Neurophysiology on Schizophrenia study |
| IQ | Intelligence quotient |
| ISA | Integrated spindle activity |
| MCCB | MATRICS Consensus Cognitive Battery |
| MST | Finger Tapping Motor Sequence Task |
| NREM | Non-rapid eye movement |
| PAD | Predicted age difference |
| PANSS | Positive and Negative Syndrome Scale |
| PC | Principal component |
| PCA | Principal component analysis |
| PSD | Power spectral density |
| PSI | Phase slope index |
| REM | Rapid eye movement |
| SCID | Structured Clinical Interview for DSM Disorders |
| SCZ | Schizophrenia |
| SD | Standard deviation |
| SO | Slow oscillation |
| SS | Slow spindle |
| TIB | Time in bed |
| TST | Total sleep time |
| UMAP | Uniform Manifold Approximation and Projection |

***Table S1 Abbreviation list***

|  | **Combined analysis** | | | | **Replication** | |
| --- | --- | --- | --- | --- | --- | --- |
| **EEG variables** | **Cluster N** | **Cluster description** | **Effect size, p-value in EEG channel with max cluster t** | | **Effect size, p-value in EEG channel with max e.s. in Wave 1** | |
|  |  |  | **Wave 1** | **Wave 2** | **Wave 1** | **Wave 2** |
| SS density | 1 | 33 chs, p=0.002 max t=-6 at P7 | e.s.=-0.8, p=8e-05 | e.s.=-0.6, p=4e-04 | e.s.=-0.8,p=8e-05 at P7 | e.s.=-0.6,p=4e-04 at P7 |
| FS density | 1 | 57 chs, p=3e-04 max t=-9 at C2 | e.s.=-1.2, p=9e-06 | e.s.=-1.7, p=3e-09 | e.s.=-1.3,p=4e-06 at FC2 | e.s.=-1.4,p=4e-09 at FC2 |
| SS amplitude | 1 | 53 chs, p=3e-04 max t=-6 at P6 | e.s.=-0.7, p=6e-05 | e.s.=-0.6, p=5e-04 | e.s.=-0.8,p=2e-05 at CP3 | e.s.=-0.5,p=2e-03 at CP3 |
| FS amplitude | 1 | 51 chs, p=3e-04 max t=-6 at C2 | e.s.=-0.6, p=5e-04 | e.s.=-0.7, p=5e-05 | e.s.=-0.6,p=7e-04 at CZ | e.s.=-0.6,p=2e-04 at CZ |
| SS ISA | 1 | 57 chs, p=3e-04 max t=-8 at C2 | e.s.=-0.9, p=4e-05 | e.s.=-1, p=2e-08 | e.s.=-1,p=2e-04 at CPZ | e.s.=-1,p=2e-07 at CPZ |
| FS ISA | 1 | 57 chs, p=3e-04 max t=-7 at F1 | e.s.=-0.7, p=5e-03 | e.s.=-1, p=1e-06 | e.s.=-0.7,p=5e-03 at F1 | e.s.=-1,p=1e-06 at F1 |
| SS duration | 1  2 | 14 chs, p=0.05 max t=4 at Fp1  21 chs, p=0.02 max t=-4 at P1 | e.s.=0.5, p=3e-02; e.s.=-0.6, p=4e-02 | e.s.=0.7, p=1e-02; e.s.=-0.3, p=6e-02 |  |  |
| FS duration | 1 | 57 chs, p=3e-04 max t=-7 at CZ | e.s.=-0.8, p=2e-03 | e.s.=-1, p=4e-07 | e.s.=-1,p=2e-03 at P5 | e.s.=-0.9,p=1e-05 at P5 |
| SS chirp | 0 | - |  |  |  |  |
| FS chirp | 1 | 31 chs, p=0.002 max t=-6 at POz | e.s.=-0.9, p=2e-04 | e.s.=-0.7, p=2e-04 | e.s.=-1.1,p=6e-04 at O1 | e.s.=-0.5,p=5e-03 at O1 |
| SS frequency | 1 | 24 chs, p=0.01 max t=-5 at FZ | e.s.=-0.6, p=1e-02 | e.s.=-0.6, p=2e-03 |  |  |
| FS frequency | 0 | - |  |  |  |  |
| SS coupl mag | 0 | - |  |  |  |  |
| FS coupl mag | 1 | 32 chs, p=0.002 max t=6 at PZ | e.s.=0.7, p=1e-02 | e.s.=0.9, p=2e-05 |  |  |
| SS coupl overlap | 1 | 57 chs, p=3e-04 max t=-8 at P3 | e.s.=-0.6, p=6e-03 | e.s.=-1.1, p=2e-09 | e.s.=-1.1,p=4e-03 at F3 | e.s.=-1,p=2e-06 at F3 |
| FS coupl overlap | 1 | 24 chs, p=0.006 max t=5 at PO4 | e.s.=0.3, p=5e-02 | e.s.=0.6, p=1e-03 |  |  |
| SS coupl angle | 1 | 35 chs, p=3e-04 max t=-7 at P7 | e.s.=-1.1, p=4e-05 | e.s.=-0.9, p=3e-05 | e.s.=-1.1,p=4e-05 at P7 | e.s.=-0.9,p=3e-05 at P7 |
| FS coupl angle | 1 | 22 chs, p=0.01 max t=-5 at TP8 | e.s.=-0.6, p=3e-03 | e.s.=-0.6, p=2e-04 | e.s.=-0.6,p=3e-03 at P4 | e.s.=0,p=4e-01 at P4 |
| SS ph-fr coupl | 1 | 49 chs, p=3e-04 max t=-5 at FC6 | e.s.=-0.7, p=3e-02 | e.s.=-1, p=4e-05 | e.s.=-0.7,p=2e-03 at C3 | e.s.=-0.4,p=1e-02 at C3 |
| FS ph-fr coupl | 1 | 8 chs, p=0.05 max t=3 at AFZ | e.s.=0, p=0.7 | e.s.=0.8, p=1e-04 |  |  |
| SO density | 1 | 56 chs, p=3e-04 max t=9 at O2 | e.s.=1.1, p=1e-05 | e.s.=1.2, p=1e-07 | e.s.=1.2,p=5e-06 at O1 | e.s.=1.3,p=4e-07 at O1 |
| SO duration | 1 | 54 chs, p=3e-04 max t=11 at CZ | e.s.=1.9, p=3e-07 | e.s.=1.9, p=2e-10 | e.s.=1.9,p=3e-07 at CZ | e.s.=1.9,p=2e-10 at CZ |
| SO slope | 1 | 55 chs, p=3e-04 max t=-9 at CZ | e.s.=-1.1, p=1e-06 | e.s.=-1, p=3e-07 | e.s.=-1.1,p=1e-06 at CZ | e.s.=-1,p=3e-07 at CZ |
| SO neg.peak amplitude | 1 | 15 chs, p=0.04 max t=-3 at FCZ | e.s.=-0.5, p=6e-02 | e.s.=-0.2, p=7e-02 |  |  |
| SO p-to-p amplitude | 1 | 14 chs, p=0.05 max t=-3 at FCZ | e.s.=-0.5, p=2e-01 | e.s.=-0.2, p=1e-01 |  |  |
| PSD | 1  2 | 720 chs, p=0.01 max t=-8 at C2 at 3Hz (1.5-5.75Hz)  848 chs, p=0.01 max t=-7 at C4 at 13.75Hz (9.5-15.75Hz) | e.s.=-1.2, p=5e-06; e.s.=-0.7, p=5e-04 | e.s.=-0.9, p=7e-07; e.s.=-0.8, p=4e-06 | e.s.=-1.2,p=3e-06 at CZ at 3.25 Hz | e.s.=-1,p=1e-06 at 3.25 Hz at CZ |
| PSI | 1  2 | 210 chs, p=3e-04 max t=-7 at AF4 at 11Hz (3-14Hz)  227 chs, p=3e-04 max t=7 at P3 at 11Hz (6-19Hz) | e.s.=-1.1, p=2e-05; e.s.=1.1, p=3e-06 | e.s.=-0.8, p=4e-05; e.s.=0.7, p=1e-04 | e.s.=1.3,p=6e-06 at P5 at 11 Hz | e.s.=0.7,p=5e-05 at P5 at 11 Hz |

**Table S2 Group differences between SCZ and CTR in the full sample (Wave 1 and Wave 2 combined)**

| **Predictor of PAD (SCZ-only joint model)** | ***N* (of 175 patients)** | ***b*** |  | ***p*** |  |
| --- | --- | --- | --- | --- | --- |
|  |  |  |  |  |  |
| Age (yrs) | . | -0.01 |  | 0.12 |  |
| Sex (M) | 101 | 0.22 |  | 0.10 |  |
|  |  |  |  |  |  |
| Olanzapine | 81 | 0.89 |  | **3E-06** | *** |
| Clozapine | 22 | 0.48 |  | **0.026** | * |
| Amisulpride | 57 | -0.19 |  | 0.25 |  |
| Quetiapine Fumarate | 13 | 0.23 |  | 0.38 |  |
| Aripiprazole | 26 | -0.09 |  | 0.65 |  |
| Risperidone | 23 | 0.05 |  | 0.84 |  |
|  |  |  |  |  |  |
| Anticholinergics | 23 | 0.57 |  | **0.0032** | ** |
| Emotion stabilizers and antiepileptics | 43 | 0.34 |  | **0.023** | * |
| Sedatives and tranquilizers | 45 | -0.32 |  | **0.030** | * |

**Table S3 Case-only joint linear model of PAD regressed on common medications, covarying for age & sex. * p < 0.05,** p < 0.01,*** p < 0.001**

| **Correlates of age model features** | **SCZ vs CTR comparisons** | | | | | | **Within SCZ comparisons** | |
| --- | --- | --- | --- | --- | --- | --- | --- | --- |
|  | ***All SCZ*** | | **Olanzapine = Y** | | **Olanzapine = N** | | **Olanzapine Y vs N (SCZ only)** | |
| **Model feature** | ***b*** | ***p*** | ***b*** | ***p*** | ***b*** | ***p*** | ***b*** | ***p*** |
|  |  |  |  |  |  |  |  |  |
| N2 theta kurtosis | **-0.24** | **8E-18** | **-2.27** | **8E-14** | **-0.82** | **7E-06** | **-0.23** | **3E-08** |
| Spindle density | **-0.19** | **1E-11** | **-1.26** | **4E-09** | **-0.80** | **2E-05** | **-0.13** | **6E-04** |
| N3 theta kurtosis | **-0.16** | **3E-08** | **-1.50** | **4E-11** | -0.31 | 0.057 | -0.20 | **5E-08** |
| N2 kurtosis | **-0.16** | **3E-08** | **-0.87** | **2E-06** | **-0.62** | **0.00013** | -0.05 | 0.24 |
| N2 alpha kurtosis | **-0.16** | **8E-08** | **-1.22** | **8E-09** | **-0.40** | **0.017** | **-0.18** | **8E-06** |
| N3 delta / alpha ratio | **-0.11** | **0.00036** | **-0.60** | **0.00077** | **-0.42** | **0.0079** | -0.03 | 0.46 |
| N3 delta / theta ratio | **-0.10** | **0.00081** | **-0.55** | **0.0016** | **-0.39** | **0.012** | -0.03 | 0.51 |
| N2 sigma kurtosis | **-0.09** | **0.0015** | **-0.60** | **0.00038** | -0.27 | 0.10 | **-0.08** | **0.04** |
| N3 delta power | **-0.09** | **0.0019** | **-0.50** | **0.0043** | **-0.40** | **0.013** | -0.02 | 0.67 |
| N3 kurtosis | 0.04 | 0.20 | 0.08 | 0.61 | 0.25 | 0.089 | -0.03 | 0.47 |
| N2 delta kurtosis | -0.02 | 0.40 | **-0.40** | **0.010** | 0.13 | 0.32 | **-0.13** | **5E-04** |
| N1 alpha power | 0.01 | 0.71 | 0.21 | 0.14 | -0.15 | 0.37 | **0.08** | **0.021** |
| Spindle coupling (overlap) | 0.00 | 0.948 | -0.28 | 0.086 | 0.17 | 0.23 | **-0.08** | **0.019** |

**Table S4 Feature-wise linear models: CTR comparisons versus a) all SCZ patients, b) SCZ patients using Olanzapine only, c) SCZ patients not using Olanzapine; fourth analysis, within SCZ, comparison by Olanzapine use. - each analysis/row performed separately; rows sorted by significance of original SCZ/CTR contrast**


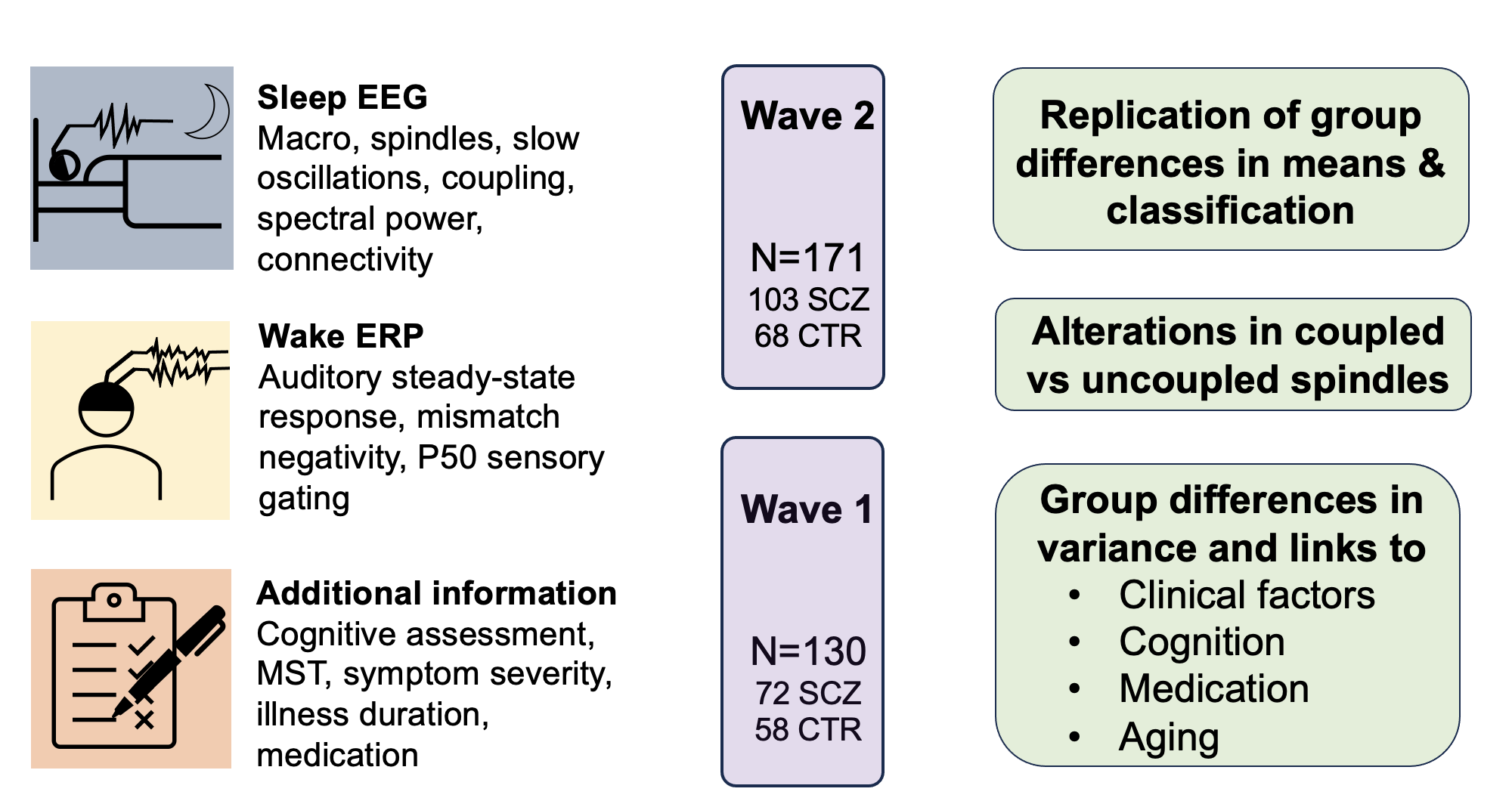


***Figure S1 Schematic illustration of the recording protocol, sample and analyses***


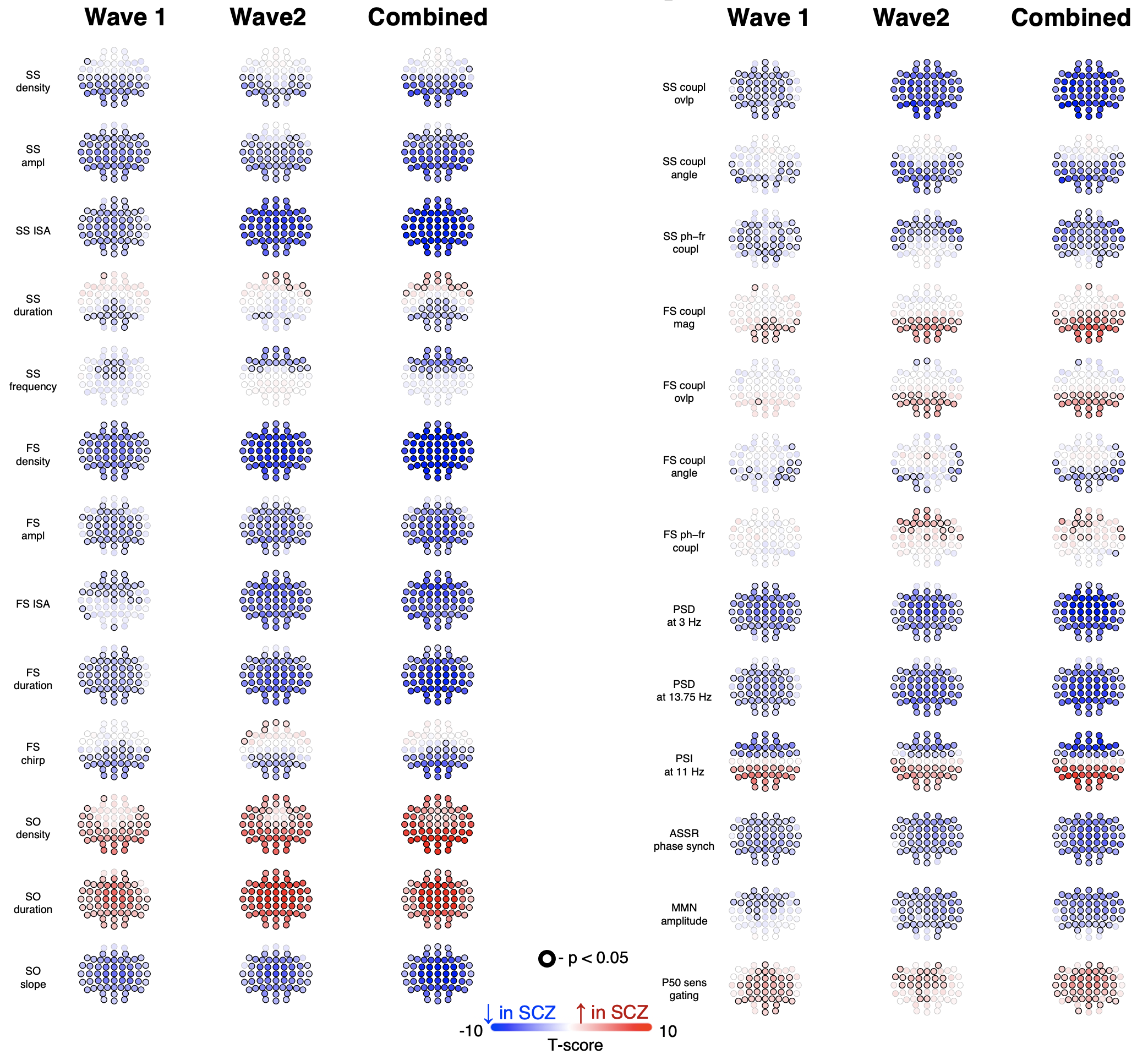
 ***Figure S2 Group differences between SCZ and CTR across waves and in the combined sample.***


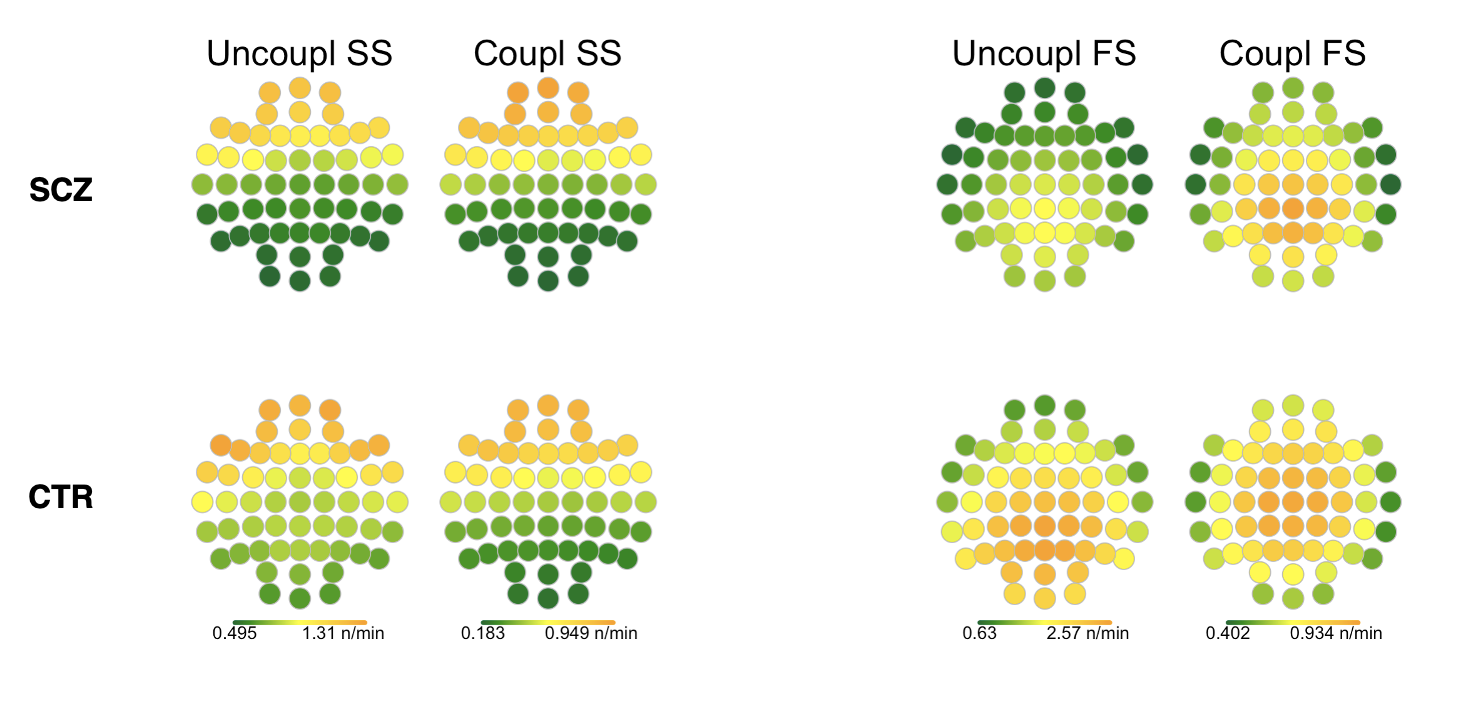


***Figure S3 Topoplots of coupled and uncoupled spindles in SCZ and CTR.*** *The topoplots represent averaged density for coupled and uncoupled spindles for each channel separately in SCZ and CTR groups.*

***Figure S4 No obvious sub-clusters within the SCZ group.*** *Visualization of the first two principal components (top row) or UMAP dimensions (bottom row) as part of attempted clustering of individuals: the left plot – PCA based on group differences between means with p < 0.001 and the right plot – PCA based on all variables with ↑ variance in SCZ with p < 0.001*


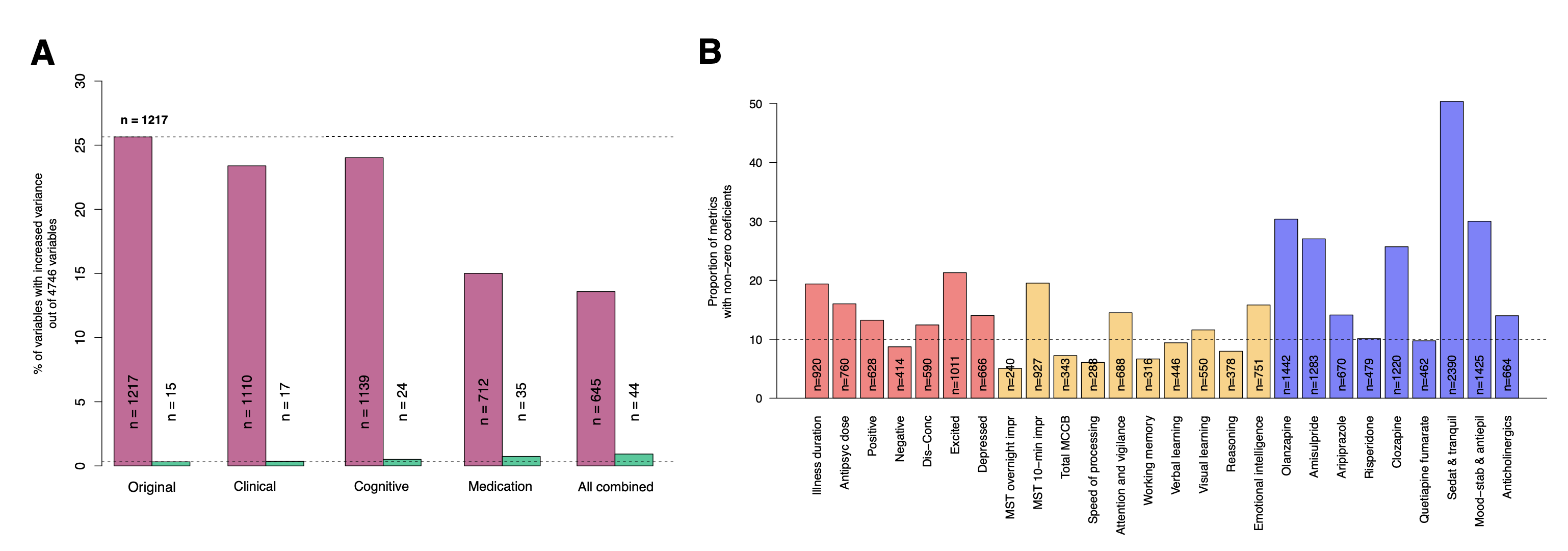


***Figure S5 Contribution of clinical, cognitive factors and medication to increased variability results.*** *A – the bar plot shows the percentage of all sleep variables still with higher variance in SCZ or CTR group after effects of clinical, cognitive factors and common medications have been simultaneously regressed out using LASSO regression for SCZ group, compared to the original estimates (horizontal dashed line); B –the bar plot illustrates the proportion of metrics for which each factor had a non-zero Lasso regression coefficient.*


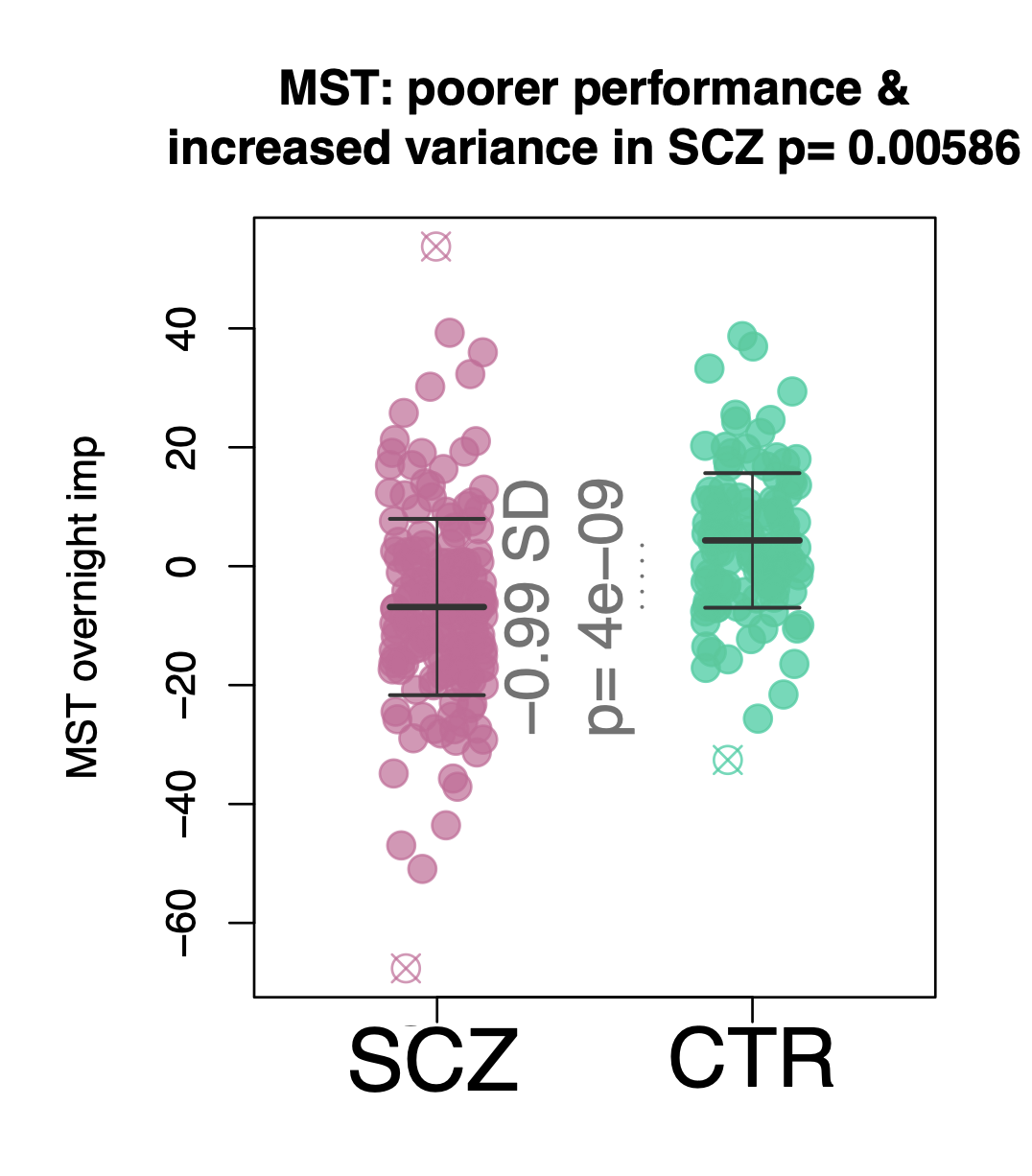


***Figure S6 Attenuation of overnight improvement of MST performance in the SCZ group versus controls.*** *Y axes represents* *the percentage increase in correct sequences from the best three training trials to the first three test trials the next morning*

***Figure S7 Clusters of significant medication tested using linear regression where each medication use is added separately as a predictor of a particular sleep metric and controlling for age and sex.*** *The medications are sorted by the number of significant clusters across N2 EEG variables.*
